# Using ddRADseq to assess the genetic diversity of in-farm and gene bank cacao resources in the Baracoa region, eastern Cuba, for use and conservation purposes

**DOI:** 10.3389/fpls.2024.1367632

**Published:** 2024-03-05

**Authors:** Angel Rafael Ramirez-Ramirez, Khaled Mirzaei, Miguel Menéndez-Grenot, Pablo Clapé-Borges, Georgina Espinosa-Lopéz, Igor Bidot-Martínez, Pierre Bertin

**Affiliations:** ^1^Faculty of Agroforestry, University of Guantánamo, Guantánamo, Cuba; ^2^Earth and Life Institute, Université catholique de Louvain (UCLouvain), Louvain-la-neuve, Belgium; ^3^Unidad de Ciencia y Técnica de Base-Baracoa / Instituto de Investigaciones Agroforestales (UCTBBaracoa / INAF), Baracoa, Cuba; ^4^Department of Biochemistry, Faculty of Biology, University of Havana, La Habana, Cuba

**Keywords:** ddRADSeq, snps, theobroma cacao, genetic diversity, Cuban cacao resources, gene bank, cacao farms

## Abstract

The Baracoa region, eastern Cuba, hosts around 80 % of the country cacao (*Theobroma cacao* L.) plantations. Cacao plants in farms are diverse in origin and propagation, with grafted and hybrid plants being the more common ones. Less frequent are plants from cuttings, TSH progeny, and traditional Cuban cacao. A national cacao gene bank is also present in Baracoa, with 282 accessions either prospected in Cuba or introduced from other countries. A breeding program associated with the gene bank started in the 1990s based on agro-morphological descriptors. The genetic diversity of cacao resources in Baracoa has been poorly described, except for traditional Cuban cacao, affecting the proper development of the breeding program and the cacao planting policies in the region. To assess the population structure and genetic diversity of cacao resources in Baracoa region, we genotyped plants from both cacao gene bank (CG) and cacao farms (CF) applying a new ddRADseq protocol for cacao. After data processing, two SNPs datasets containing 11,425 and 6,481 high-quality SNPs were generated with 238 CG and 135 CF plants, respectively. SNPs were unevenly distributed along the 10 cacao chromosomes and laid mainly in noncoding regions of the genome. Population structure analysis with these SNP datasets identified seven and four genetic groups in CG and CF samples, respectively. Clustering using UPGMA and principal component analysis mostly agree with population structure results. Amelonado was the predominant cacao ancestry, accounting for 49.22 % (CG) and 57.73 % (CF) of the total. Criollo, Contamana, Iquitos, and Nanay ancestries were detected in both CG and CF samples, while Nacional and Marañon backgrounds were only identified in CG. Genetic differentiation among CG (*F_ST_* ranging from 0.071 to 0.407) was higher than among CF genetic groups (*F_ST_*: 0.093–0.282). Genetic diversity parameters showed similar values for CG and CF samples. The CG and CF genetic groups with the lowest genetic diversity parameters had the highest proportion of Amelonado ancestry. These results should contribute to reinforcing the ongoing breeding program and updating the planting policies on cacao farms, with an impact on the social and economic life of the region.

## Introduction

The cacao species *Theobroma cacao* L is the center of the chocolate industry, involving millions of people around the world, from small farmers in remote areas of developing countries to chocolate shops in big cities of the industrial world (CacaoNet, 2022). The origin of *T. cacao* has been set in Upper Amazon, South America, in the current borders between Brazil, Colombia, Ecuador, and Perú, from where it would have been extended first to Mesoamerica during pre-Columbian times and later to other tropical and subtropical regions of Latin America, Africa, and Asia (Motamayor et al., 2008; Zhang and Motilal, 2016). Cacao bean global production was estimated at 5.76 million tons in 2020, with Côte d’Ivoire, Ghana, and Indonesia as the biggest producing countries (FAOSTAT, 2023).

In the past decade, cacao-producing areas have increased worldwide from 9.6 to 12.3 million hectares (ha) using cacao types with low cocoa quality in most cases. Despite this expansion, yield growth was minimal, with an increase from 450 kg/ha to 467 kg/ha recorded (FAOSTAT, 2023). Cacao genetic studies have a role to play in improving the yield and cocoa quality, especially when there is evidence of a narrow genetic base used in cacao farming (Zhang and Motilal, 2016; Cornejo et al., 2018). A very wide range of molecular markers has been used in cacao genetic studies (Motilal et al., 2017), which, combined with morphological data, have served for genetic diversity assessment, clone classification, QTL identification, association studies, linkage map, etc. However, more efforts are required to face future challenges of the cacao and chocolate industries, which comprise increasing demand for chocolate, including those with superior qualities, the spreading of diseases and pests, changing environmental conditions, and the need for sustainable production (Zhang and Motilal, 2016; Wickramasuriya and Dunwell, 2018).

Three traditional cacao groups have first been recognized based on plant morphological profiles: Criollo, Forastero, and Trinitario. The productivity of Criollo is low but of high quality, while Forastero is highly productive but of lower quality. Trinitario appeared as the result of the crossing of both former groups, carrying intermediate characteristics (Figueira et al., 1994; Badrie et al., 2015). More recently, a new classification system was established based on Simple Sequence Repeat (SSR) markers, and 10 cacao ancestry genetic groups have been recognized: Amelonado, Contamana, Criollo, Curaray, Guiana, Iquitos, Marañón, Nanay, Nacional, and Purús (Motamayor et al., 2008). Both classification systems are currently in use, and cacao genetic studies are challenging when it comes to the proper classification of cacao clones, especially those plants resulting from the crossing of already admixed parents (Motilal, 2018; dos Santos Menezes et al., 2022).

The use of single nucleotide polymorphism (SNP) markers to assess genetic diversity in *T. cacao* has sharply increased in the last few years. Most of the studies are based on SNP datasets derived from the study of Argout et al. (2008). These SNPs have been successfully used to describe cacao ancestry genetic group classification and genetic diversity evaluation (Ji et al., 2013; Fang et al., 2014; Cosme et al., 2016; Lindo et al., 2018; Arevalo-Gardini et al., 2019; Wang et al., 2020 and Li et al., 2021), but difficulties have been reported in the proper separation of some cacao ancestry genetic groups (Lukman et al., 2014; Takrama et al., 2014; Osorio-Guarin et al., 2017), driving the search for other SNP sets suitable for cacao classification (Mahabir et al., 2020; Gutiérrez et al., 2021).

Few studies describe the use of large SNP datasets derived from next-generation sequencing (NGS) technologies in cacao genetic diversity evaluation. Cornejo et al. (2018) sequenced 200 cacao genomes to explore cacao domestication history, and Osorio-Guarin et al. (2018) reported a modified GBS approach with 30 samples and the same references as Cornejo et al. (2018) for the identification of 7,009 SNPs carrying cacao ancestry information. Recently, GBS experiments based on genomic digestion with two enzymes were used to perform genetic studies in cacao from French Guiana, Martinique, and Colombia (Lachenaud et al., 2018; Adenet et al., 2020; Osorio-Guarin et al., 2020).

Double-digest Restriction-Associated DNA sequencing (ddRADseq) is a RADseq-derived technique that uses NGS technology to uncover hundreds or thousands of polymorphic genetic markers across the genome. This is a reduced-representation genome sequencing method that also combines two restriction enzymes to digest DNA and has become a frequently used approach for SNP marker discovery and genotyping of nonmodel organisms (Peterson et al., 2012; Andrews et al., 2016). Several ddRADseq protocols have been exploited for crop genetics studies, differing mainly in the enzyme combination and size of the selected DNA fragments. ddRADseq outperforms other GBS protocols used for cacao genetic analysis in higher average coverage and lower missing data, though it is acknowledged that the success of ddRADseq protocols requires high-quality DNA preparations (Scheben et al., 2017).

Cuba is a small cacao producer with 1,577 tons of cacao beans obtained in 2020 (ONEI, Oficina Nacional de Estadística e Información, 2021; FAOSTAT, 2023). There is a debate about the date and place of cacao introduction in Cuba, pointed either to the central region in *Mi Cuba* near Cabaiguán in 1540 from México or by French colonists running from the Haitian Revolution in late eighteenth century who settled down in the region of *Ti Arriba* in eastern Cuba (Nuñez-González, 2010). During the nineteenth century, cacao plantations and plants for self-consumption could be found in several regions of the country, including locations near Havana in the western part of the country. Reports attest to the exportation of 1,500 tons of cacao in the early 90s of the nineteenth century and 2,000 tons of production at the beginning of the 1900s, after a devastating war period during 1895–1898. Little information about the origin of the plants is available, though the harvesting of good quality cacao beans of the types “Criollo” or “Cubano”, “Guayaquil” (Ecuador), and “Caracas” (Venezuela) is recognized.

The expansion of the sugar industry during the late nineteenth and early twentieth centuries pushed cacao planting areas to places not suitable for sugar cane cropping. The mountainous regions in the eastern and central part of the country were the most appropriate, including the current provinces of Sancti Spiritus, Cienfuegos, and Villa Clara in the center and Guantánamo, Santiago de Cuba, and Granma in the east. This distribution has remained almost the same until the present, where Baracoa municipality, in Guantánamo province, excels in the favorable climate conditions for cacao farming. Baracoa hosts around 80% of Cuban cacao plantations, and more than 20% of the cultivated land in the region is used for cacao cropping, which was responsible for 74.6% of Cuban cacao production in 2020. In this region, the cacao and chocolate agroindustry is part of a strong tradition lasting decades with a great impact on both the social and economic lives of the local inhabitants (Nuñez-González, 2010; ONEI, Oficina Nacional de Estadística e Información, 2021).

In Cuba, cacao is cultivated organically, and farms use an agroforestry, multispecies, and multilayer cultivation system with shade trees and various associated perennial and annual crops. Plantations contain cacao plants of diverse origins and reproduction modes. The most abundant are grafted plants obtained from specific clones, mainly from the United Fruit Company (UF) introduced in the country around 1955. Another type is hybrid plants grown from seeds, which could be either certified seeds produced by hand pollination of certain cacao clones at Unidad de Ciencia y Técnica de Base-Baracoa, Instituto de Investigaciones Agroforestales (UCTB-Baracoa/INAF) or seeds produced on farms by farmer-selected plants under open pollination conditions. Other less common sources of cacao plants include cuttings from highly productive plants or cacao clones and progeny from Trinidad Selected Hybrids (TSH) imported as seed during the 1970s (Márquez-Rivero and Aguirre-Gómez, 2008; Nuñez-González, 2010; Martínez-Suárez et al., 2016; Menéndez-Grenot et al., 2016).

Bidot Martínez et al. (2015) analyzed another class of plants found in Cuban cacao farms, known as traditional Cuban cacao, which represents around 6% of the Cuban cacao. These are very old plants remaining in cacao plantations whose propagation has relied exclusively on farmers and are supposed to be the closest ones to the cacao primarily introduced in Cuba. These cacao plants from central and eastern Cuba were sampled, and the population structure and genetic diversity were analyzed with SSR markers and morphological descriptors. Two groups of plants were identified, mostly corresponding with the geographical regions of collection: central and eastern. The cacao ancestry of these plants was mainly divided into Amelonado, Criollo, Marañon, and Contamana. Morphological profiles revealed the Trinitario type as the most abundant among the studied plants. The persistence of these plants proves their ability to resist local environmental conditions, including diseases and pests, and in some cases, they contain seeds with white cotyledons (Bidot Martínez et al., 2017). The real productive potential of these plants is yet to be studied; however, some of them, mainly with white cotyledons, were selected for conservation purposes. A deeper knowledge of the genetic diversity of commercial cacao farms in Baracoa is crucial to facing the current and future challenges of the local cacao agroindustry and requires more in-depth studies.

A national cacao gene bank (CG) started to grow in the 1980s under the supervision of UCTB-Baracoa/INAF for conservation and research purposes. Currently, the gene bank hosts 282 accessions: 194 prospected in Cuba in the provinces of Guantánamo (163), Santiago de Cuba (24), and Mayabeque (seven), and 88 introduced from different geographical regions (South America (41), Central America (18), Caribbean (17), North America (11), and Africa (one)). The 194 prospected accessions were plants collected in field expeditions, including the traditional Cuban cacao plants aforementioned, and hybrid plants selected from breeding experiments using hand pollination between clones of interest. The 88 introduced accessions comprise cacao clones of the series UF, Pound, SCA, EET, ICS, TSH, GS, SIAL, and SIC—among others—imported from other countries throughout the second half of the last century.

Several of these accessions have been partially characterized with morphological and agronomical descriptors, including resistance to *Phytophthora palmivora* and commercial quality, as part of a breeding and selection program launched by UCTB-Baracoa/INAF during the 1990s (Menéndez-Grenot et al., 2012, 2014; Martínez-Suárez et al., 2016; Matos-Cueto et al., 2016; Menéndez-Grenot et al., 2016). This program has allowed the identification of clones with high productive potential and the establishment of procedures for certified hybrid seed production from selected clones. Unfortunately, genetic characterization of the gene bank is pending, and the program goal for higher quality cacao in terms of cocoa and chocolate quality remains evasive. Reversing such a scenario is mandatory in the efforts to get access to more demanding cacao markets.

The genetic characterization of plants of the Cuban National cacao gene bank (CG) and cacao farms (CF) from Baracoa region, including the identification of ancestry genetic groups, represents a significant step forward for the future development of the cacao and chocolate industries in the region. To this end, the goals of our study were (1) to apply a new ddRADseq protocol for cacao SNP identification and use these SNPs and (2) use these SNPs to assess the population structure and genetic diversity of conserved and in-use cacao resources in the Baracoa region. The results derived from this research provide the much-needed information about the genetic diversity of cacao resources in Baracoa. This information could allow a better planning of breeding experiments as part of the ongoing breeding and selection program and the revalorization of previously obtained cacao clones, especially when association studies using phenotypic data already collected will be possible. Additionally, an evaluation of the cacao ancestry genetic groups to be introduced in both the national gene bank and cacao farms will be possible, resulting in an improvement in the genetic diversity of conserved and in-used cacao resources.

## Material and methods

### Plant material

Mature leaves were collected from clone accessions of *Theobroma cacao* of the CG. In order to select the samples from CF, a survey was applied to cover the diversity of cacao farms currently in production (commercial cacao farms) present in Baracoa with the help and experience of cacao specialists from the UCTB-Baracoa/INAF. The farms were located in the three major productive poles: Jamal, San Luis, and Paso de Cuba/Sabanilla, and the survey covered production (yield of cacao and other side products), soil properties (fertility, humidity, drainage, erosion), topography, slope orientation, canopy diversity, and more importantly, cacao plant origin according to farmers (grafted, hybrid, traditional).

Four farm types were identified: type 1 farms consisted of flat, wet valleys with high humidity and variable soil drainage, with mostly hybrid and grafted cacao plants; type 2 contained mostly flat and wet valleys with favorable soil drainage but only grafted cacao plants; type 3 contained mountainside farms with favorable drainage and eroded soil with hybrid and traditional cacao plants; and type 4 was a mixture of flat and mountainside topography with favorable soil drainage and combined the three cacao plants’ origins: traditional, grafted, and hybrids.

Seven cacao farms, comprising all farm types identified, were selected as representative of the surveyed ones (**Supplementary Table S1**). On these seven farms, 32 plots with 25 plants each were randomly raised and established as sampling units. All combinations of cacao plant origins and topographic conditions detected on each farm were covered by the plots. The total number of plots included replica plots that were done whenever the farm size allowed it. Plants were numbered from 1 to 800, and 160 of them were randomly taken (five per plot) with a random number generation procedure. Mature leaves of selected plants were collected for analysis.

Leaves of the sampled plants were placed in a closed container with an air dehumidifier to ensure fast drying; the temperature was regularly monitored and always maintained below 40°C. Once dried, the leaves were kept at −70°C until use.

Cacao plants (65) belonging to the 10 cacao ancestry genetic groups, according to Motamayor et al. (2008) were taken as reference plants. Cornejo et al. (2018) determined the cacao ancestry of these plants using whole genome sequencing experiments and classified them as Amelonado (10), Contamana (seven), Criollo (four), Curaray (five), Guiana (seven), Iquitos (six), Marañon (10), Nacional (four), Nanay (eight), and Purús (four) (**Supplementary Table S2**).

### DNA extraction and purification

DNA was purified following a previously described protocol with some modifications (Souza et al., 2012). A sample of 25 mg of dry cacao leaves was frozen with liquid nitrogen and reduced to a fine powder using a Tissue Lyser (QIAGEN, Germany). The powder was washed twice with 1.5 mL of cold sorbitol buffer (0.35 M sorbitol, 100 mM Tris-HCl, 5 mM EDTA, 1% PVP-40, 1% 2-mercaptoethanol, pH = 8.0) and centrifuged for 10 min at 4,500×*g* and 4°C. The pellet obtained was resuspended in 1 mL of prewarmed (65°C) extraction buffer (3% CTAB, 100 mM Tris-HCl, 20 mM EDTA, 3 M NaCl, pH = 8.0); additionally, 20 µL of proteinase K (10 mg/mL), 35 µL of 30% sarkosyl, and 30 mg of PVPP were added to each tube. The homogenate was incubated at 65°C for 1 h and mixed by inversion every 15 min. After cooling at room temperature, 800 µL of chloroform:isoamyl alcohol (24:1) was added and mixed by inversion for 15 min, followed by a centrifugation step at 13,000×*g* for 10 min at room temperature. The upper phase was transferred to a fresh clean tube and volumes equal to 0.1 times of 3 M of NaAc at pH 5.2 and 2/3 times of cold isopropanol (−20°C) were added to the homogenates. Tubes were mixed by inversion and kept at −20°C overnight. The DNA pellet was collected by centrifugation at 15,500×*g* for 30 min at 4°C, washed by the addition of 700 µL of 70% ethanol, and centrifuged again for 5 min, as previously. The supernatants were carefully removed to avoid losing the nucleic acid pellet, and the tubes were left open to dry at room temperature. Pellets were resuspended in 100 μL of TE buffer with 2 µL of DNase-free RNase A (10 mg/mL) by incubation at 37°C until complete dissolution and avoiding pipetting. DNA preparations were stored at −20°C until required.

A DNA cleaning step was implemented using the silica columns provided with the DNeasy Plant Pro Kit from QIAgen (2019) and following the manufacturer’s instructions with some modifications. Shortly, 200 µL of Milli-Q distilled water was added to 100 µL of the purified DNA solution. The mix was placed in a water bath at 37°C until a homogeneous solution was achieved; next, 375 µL of the APP buffer was added, and the homogenate was applied to the silica columns. Column washing steps were completed according to the manufacturer. DNA was eluted in 65 µL of TE buffer and kept at −20°C until use.

DNA integrity was checked by agarose gel electrophoresis at 1.5%. DNA yield was estimated with a fluorimeter and fluorescent DNA-binding dye (Qubit™ dsDNA BR Assay Kit, Thermo Fisher Scientific, USA), according to the manufacturer’s instructions. A total of 406 samples were successfully purified: 264 from CG and 142 from CF, which were used for library preparation.

### ddRADseq library preparation and sequencing

Reagents used in library preparation were obtained from New England Biolabs (NEB), USA unless specified. ddRADseq libraries were prepared as described (Peterson et al., 2012) with some modifications. Briefly, 1,000 ng of DNA were digested with 10 U of EcoRI HF and 5 U of NlaIII using the Cut Smart Buffer in a final volume of 30 µL. The digestion reactions were left to occur at 37°C overnight. A volume of 15 µL of digested DNA was put to ligation with adaptors designed for ddRADseq sequencing libraries (Peterson et al., 2012) using T4 ligase (0.1 U per reaction). Ligation reactions occurred for 8 h at 16°C in a final volume of 20 µL.

After ligation, samples were combined to form pools containing between 44 and 48 samples (sublibraries). The sublibraries were purified with magnetic beads (Promega, USA) and fragments between 300-500 bp were selected using a BluePippin instrument (Sage Science Inc., USA). Size-selected sublibraries were polymerase chain reaction (PCR)-enriched using the enzyme Phusion^®^ High-Fidelity DNA Polymerase (NEB) with producer recommendations. Reactions occurred for 12 cycles, and sublibraries indexes were added according to Peterson et al. (2012). PCR products were magnetic bead-purified and combined to conform three ddRADseq libraries. Libraries were sequenced on a HiSeq2500 instrument (Illumina, San Diego, CA, USA) following a pair-end strategy with a read length of 150 bp.

### Data processing and SNP calling

DNA sequence quality was checked with FastQC v0.11.9. Demultiplexing was done with process_radtags from Stacks v2.5 (Catchen et al., 2013) following recommended options (Rochette and Catchen, 2017). Sequences with an average base quality (*Q*) score lower than 25 in a single 15-nt window, following a sliding window algorithm, were discarded. After that, TrimGalore/cutadapt was employed to remove 8 nt and 15 nt from the 5′ and 3′ ends, respectively, along with a 3′ quality trimming to remove bases with *Q* < 25 (Krueger, 2017). Only paired reads longer than 75 nt were kept.

Sequences were aligned to the Matina 1–6 cacao reference genome (Motamayor et al., 2013) using the BWA MEM algorithm (BWA v0.7.17) with the default settings (Li and Durbin, 2009). *sam* files were converted into *bam* files with Samtools v1.10 (Li et al., 2009), and output files were cleaned, fixed, sorted, and the RG group was added with Picard tools v2.18.25 (Broad Institute, 2016).

For SNP calling, samples from the cacao CG and CF were analyzed independently from each other, and only the read mapping in cacao chromosomes was used. This approach was applied to properly assess the genetic diversity in both CG and CF scenarios, especially when plant origins were different. Indeed, gene bank included both Cuban prospected and worldwide imported clones, while farm plants included locally propagated plants by different methods. SNPs were identified with GATK v4.2.0.0 (Van der Auwera and O’Connor, 2020), combining the following tools: BaseRecalibrator, HaplotypeCaller, CombineGVCFs, and GenotypeGVCFs. Raw SNPs were filtered following GATK hard filtering recommendations: QD < 2.0, SOR > 3.0, MQ < 50.0, FS > 50.0, MQSumRank < −12.5 and ReadPosSumRank < −8.0 (Caetano-Anolles, 2022). An additional filtering for representativeness was applied using VCFtools v0.1.16 (maf > 0.05, site missing < 5%, biallelic, SNP depth coverage: 10–80, SNP spacing > = 1,000 nt) (Danecek et al., 2011).

Final SNP datasets were obtained by intersecting filtered SNPs from CG and CF samples with another SNP dataset built—as described (Cornejo et al., 2018)—from available sequence data of 65 cacao reference plants of the 10 ancestry genetic groups described by Motamayor et al. (2008) (reference SNP dataset, **Supplementary Table S2**), keeping only the intersected SNPs (coincident). These final SNP datasets (henceforth SNP datasets) from CG and CF samples contained 11,425 and 6,481 variants, respectively, and were employed for further analysis. SNP datasets, Transition/Transversion ratios, missing data, and depth of coverage on individual bases were estimated with VCFtools. The SNP distribution along the cacao chromosomes was analyzed using a 1 Mb window size with the function CMplot from the R package with the same name (v4.5.0) (Yin et al., 2021).

### SNP annotation and gene ontology analysis

SNPs from CG and CF were annotated separately with SnpEff software v5.1d (Cingolani et al., 2012) using the available annotation for the *Theobroma caca*o Matina 1–6 genome. The potential effect of the SNPs on gene expression and function, considering SNP position with respect to coding regions, was analyzed.

PANTHER classification system version 17.0 (released 22 February 2022) (http://pantherdb.org/) was used for gene ontology analysis as described (Mi et al., 2019). For that purpose, genes containing SNPs with moderate and high impact, according to the SnpEff tool, were selected. Gene lists were analyzed against the following databases: GO-Slim Molecular Function, GO-Slim Biological Process, and PANTHER Protein Class (GO Consortium C, 2017; Mi et al., 2019). Overrepresentation analyses of the identified genes with the databases GO molecular function complete and GO biological process complete were executed based on Fisher’s exact test (*p* < 0.05) with false discovery rate correction (FDR < 0.05).

### Population analysis

#### Population structure

Two types of population analysis were undertaken: the first one without reference plants in order to detect genetic groups, both among CG and CF plants, independently from each other; the second one with reference plants to identify the membership of both CG and CF plants to genetic groups of cacao according to Motamayor et al. (2008), here referred to as ancestry genetic groups.

Firstly, CG and CF genetic groups were detected by ADMIXTURE v1.3.0 software (Alexander et al., 2009) with a fivefold cross-validation procedure under penalized (−*l* 500, −*e* 0.2) and random seedling (−s time) conditions (Alexander and Lange, 2011; Cornejo et al., 2018). Twenty replicas for *K* values ranging from 1 to 18 (CG) and from 1 to 12 (CF) were performed following the recommendations of Liu et al. (2020). The best *K* value identification was guided by the premise that all identified genetic groups must contain individuals with a high membership (*Q* > 0.90). To this end, *Q*-matrices were analyzed both individually and by the online platform CLUMPAK (Kopelman et al., 2015), along with the cross-validation error (CV error) and the number of iterations to convergence of ADMIXTURE runs (Alexander et al., 2009; Alexander and Lange, 2011). Secondly, the kinship to the cacao ancestry genetic groups of Motamayor et al. (2008) of each individual was calculated by running ADMIXTURE in supervised mode with the aforementioned penalized options. Shortened versions of the reference SNP dataset containing the same SNP positions as CG and CF SNP datasets were used for training purposes of ADMIXTURE runs under supervised mode. The two vcf files used for cacao ancestry estimation with ADMIXTURE contained: 1° CG plants and the cacao reference plants and 2° CF plants and the cacao reference plants, and were obtained by properly merging CG and CF SNP datasets and the shortened reference SNP datasets abovementioned. The capacity of SNPs included in CG and CF SNP datasets to properly separate the 65 reference plants into the expected cacao ancestry genetic group was assessed before vcf files merged, and shortened reference SNP datasets were used for that purpose (**Supplementary Tables S2–S6**; **Supplementary Figures S1–S6**).

#### Clustering and PCA

Both CG and CF samples were studied alone and in combination with the reference clones of the cacao ancestry genetic groups, leading to four different clustering and PCA analyses. Clustering analyses were performed by unweighted pair-group method with arithmetic averages (UPGMA) from Hamming distance matrices. Trees were built using a bootstrapping procedure with 1,000 replicas with aboot function of poppr package v2.9.4 (Kamvar et al., 2014, 2015) from R statistical language version 4.2 (R Core Team, 2020) and visualized with ggtree package v3.10.0 (Yu et al., 2017). For principal component analysis (PCA), the glPca function from adegenet package v2.1.10 (Jombart, 2008; Jombart and Ahmed, 2011) was used with the number of alleles scaled and the alleles assumed as a unit.

#### Differentiation between groups and genetic diversity

An admixed group (Adm) of plants was formed in ADMIXTURE (see Results) and was excluded from these analyses. AMOVA test was used to detect differences among the genetic groups defined by ADMIXTURE, according to Excoffier et al. (1992), with the function poppr.amova from poppr package v2.9.4. For the CF samples, additional putative levels of variability were also considered, i.e., farms and productive poles of the plants under study. The significance of the test was estimated by the randtest function of ade4 package v1.7-22 (Dray and Dufour, 2007; Thioulouse et al., 2018) with 999 permutations (R Core Team, 2020).

*F*_ST_ pairwise coefficients for the ADMIXTURE-defined groups were estimated according to Weir and Cockerham (1984) with the gl.fst.pop function of dartR package v2.9.7 (Gruber et al., 2018; Mijangos et al., 2022); 10,000 bootstrappings were performed for confidence intervals (95%) and *p*-value estimation. Genetic diversity parameters, i.e., observed (*H*_obs_) and expected (*H*_exp_) heterozygosity, and the polymorphic information content (PIC) were estimated with adegenet and poppr packages from the R program.

## Results

### SNP calling and SNP dataset characterization

The three ddRADseq libraries prepared contained 406 different cacao plants, and 1,806,293,684 reads were generated during DNA sequencing. After data cleaning, 1,244,420,354 DNA sequences were retained, making an average of 2,941,892 reads per sample. However, 33 samples were removed as their read counts dropped below 1 million reads. Thus, in total, 373 cacao plants were properly sequenced with the described protocol: 238 from the CG and 135 from the CF.

The reads were aligned to the Matina 1–6 cacao reference genome (Motamayor et al., 2013). CG and CF plants were treated independently from each other for the SNP calling process to properly assess the genetic diversity of gene bank and in-farm cacao resources. Raw SNPs were estimated in 1,707,351 for CG and 731,688 among CF plants. After filtering with VCFtools, 13,418 and 7,655 SNPs were retained for CG and CF, respectively. These SNPs were intersected with the reference SNP dataset of plants of the cacao ancestry genetic groups described by Motamayor et al. (2008) (see Material and methods), retaining only intersected SNPs. The resulting datasets had 11,425 and 6,481 SNPs for the CG and CF plants, respectively, and were used for further analysis.

The distributions of the variables used during GATK hard filtration had similar profiles for both CG and CF SNP datasets, and the bell-shaped curve obtained for some of them suggested a low or absence of bias in the data supporting the identified SNPs (**Supplementary Figure S7**). The transition/transversion ratios estimated by VCFtools software showed comparable values among the SNP datasets (**Supplementary Table S7**). The means of missing data per individual were 1.81% (CG) and 1.54% (CF), but still 11 samples had missing data higher than 10%, with 17.1% being the highest. The average coverage depth per sample for both SNP datasets was 20.5×, and in 12 cases, depth dropped below 10×, reaching 6× in one of them. Plants with relatively high missing data and low coverage were not removed from the datasets because they accounted for less than 3% of the total, and studies using up to 50% of individual missing data threshold have been reported in cacao genetic studies based on SNP markers (Adenet et al., 2020; Gutiérrez et al., 2021).

**Figure 1:**
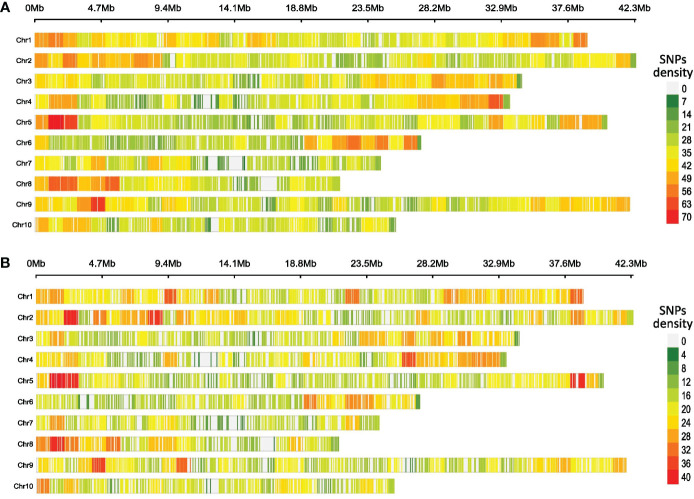
Heatmap of SNP density per chromosome of cacao gene bank (CG) **(A)** and cacao farm (CF) **(B)** SNP datasets. A 1-Mb window was set for counting and plotting purposes. The plots were generated using the R package CMPlot.

The average SNPs per Mb were 34.6 and 19.6 for CG and CF SNP datasets, respectively. The SNP markers were distributed throughout the cacao genome, and SNP densities increased as windows moved from the center of the chromosomes to the telomeres (**Figure 1**). For both cases, chromosome 1 had the higher average density per Mb (CG = 38.9 and CF = 22.2) while chromosome 7 showed the lowest values (CG = 28.9 and CF = 16.9).

**Figure 2:**
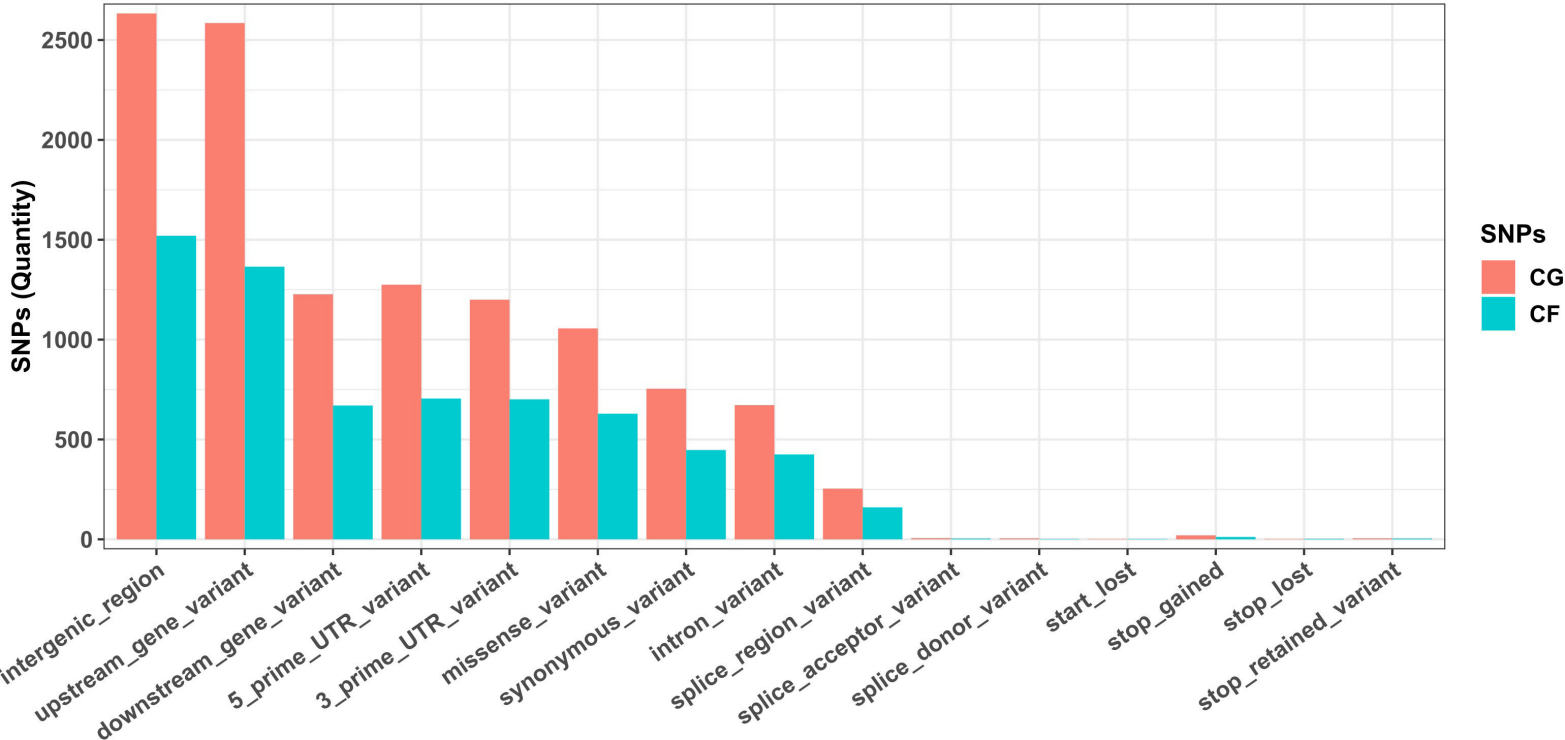
Annotation of SNPs from Cuban cacao gene bank (CG) and Cuban cacao farm (CF) SNP datasets, analyzed independently with SnpEff software, using the available annotation for the *Theobroma caca*o Matina 1–6 genome as a reference.

Most SNPs detected in both CG and CF SNP datasets lay on noncoding regions of the cacao reference genome, since the combination of variant categories “intergenic region”, “upstream/downstream gene”, and “UTR and intron” accounted for 83.96% of CG SNPs and 83.10% of CF SNPs. Missense and synonymous variants were 1,047 (9.16%) and 754 (6.60%) CG SNPs, and 625 (9.64%) and 447 (6.90%) CF SNPs, respectively. Low amounts of start/stop and splice-related variants were also detected (**Figure 2**).

For ontology analyses, lists of genes containing SNPs with moderate and high impact were built for each dataset. SNPs included in these impact categories had already been classified by SnpEff software as missense, splice_donor/acceptor, start_lost, stop_gained, and stop_lost variants. The lists built contained 1,052 and 636 genes for the CG and CF SNP datasets, respectively. Cellular and metabolic processes and biological regulation were the GO-Slim Biological Process terms with the highest number of hits (**Supplementary Figure S8**). In the case of GO-Slim Molecular Function, catalytic activity, binding, and transporter activity were the most abundant ones, and the protein class categories with the highest counts were metabolite interconversion enzyme, protein-modifying enzyme, transporter, and transmembrane signal receptor among the analyzed gene lists (**Supplementary Figure S8**).

Overrepresentation tests for molecular function and biological process (GO complete) of the gene lists revealed a more than expected representation of genes involved in protein kinase, ATP-related, and carbohydrate-binding activities for GO molecular function complete; while protein phosphorylation was the only biological process overrepresented (GO complete) (**Table 1**).

**Table 1:** Overrepresentation test results of genes containing moderate- and high-impact SNPs from CG and CF SNP datasets.

GO molecular function complete	REF No.	INPUT No.	Expec	Fold enrich	+/−	Raw *p*-value	FDR
Cacao gene bank SNPs/genes							
Transmembrane receptor protein serine/threonine kinase activity	43	8	1.56	5.12	+	3.70*E*−04	2.64*E*−02
Protein serine kinase activity	83	11	3.01	3.65	+	4.51*E*−04	3.13*E*−02
ABC-type transporter activity	110	16	3.99	4.01	+	8.90*E*−06	7.94*E*−04
ATP binding	2,127	160	77.24	2.07	+	2.25*E*−17	1.40*E*−14
ADP binding	94	12	3.41	3.52	+	3.44*E*−04	2.52*E*−02
Carbohydrate binding	289	27	10.49	2.57	+	3.34*E*−05	2.69*E*−03
Metal ion binding	2,869	145	104.18	1.39	+	8.55*E*−05	6.47*E*−03
GO biological process complete							
Protein phosphorylation	1,160	95	42.12	2.26	+	1.82*E*−12	4.23*E*−09
Cacao farm SNPs/genes							
Protein serine/threonine kinase activity	697	36	15.3	2.35	+	6.27*E*−06	6.26*E*−04
ATP-dependent activity	676	30	14.84	2.02	+	5.02*E*−04	4.47*E*−02
ATP binding	2,127	90	46.7	1.93	+	4.71*E*−09	1.47*E*−06
Carbohydrate binding	289	19	6.34	2.99	+	4.41*E*−05	4.24*E*−03
GO biological process complete							
Protein phosphorylation	1,160	53	25.57	2.08	+	1.29*E*−06	9.61*E*−03

Overrepresented GO complete terms according to the PANTHER classification system. Only terms/categories with significant results (p < 0.05) and with false discovery rate (FDR < 0.05) are shown. REF No., counting for the term/category in the reference; INPUT No., counting for the category/term in the input list; Expec, expected counting for the category/term; Fold Enrich, enrichment fold for the category/term; “+/−” indicates if the category/term is overrepresented (+) or underrepresented (−).

The new ddRADseq protocol used for cacao SNP genotyping, which proved to be efficient for high-quality SNP identification using samples from the gene bank and commercial cacao farms in the Baracoa region. The 11,425 and 6,481 variants contained in the CG and CF SNP datasets were spread throughout the 10 cacao chromosomes. Most of these SNPs were laid on noncoding regions of the cacao genome and biases toward ATP and protein phosphorylation-related activities were supported by the overrepresentation tests performed.

### Population structure analysis of the Cuban cacao gene bank and cacao farms

The identification of the number of clusters or genetic groups (*K*) among the 238 samples of the CG proved to be a difficult task because no clear minimum for cross-validation error (CV error) was achieved when ADMIXTURE runs were analyzed, contrary to CF samples (**Supplementary Figure S9**) (Alexander et al., 2009; Alexander and Lange, 2011). Since we were seeking a scenario in which all identified genetic groups must contain at least some individuals with a high membership (*Q* > 0.9), *Q*-matrices from every ADMIXTURE run of all *K* values assessed were analyzed. With CG plants, this premise of high membership groups was consistently fulfilled until *K* = 6, where samples with high kinship (*Q* > 0.9) for all identified groups were found in 17 out of 20 *Q*-matrices (**Supplementary Table S8**). With *K* = 7, nine *Q*-matrices matched the premise; with *K* = 8 only three; this value keeps decreasing until zero for *K* ≥ 11.

**Figure 3:**
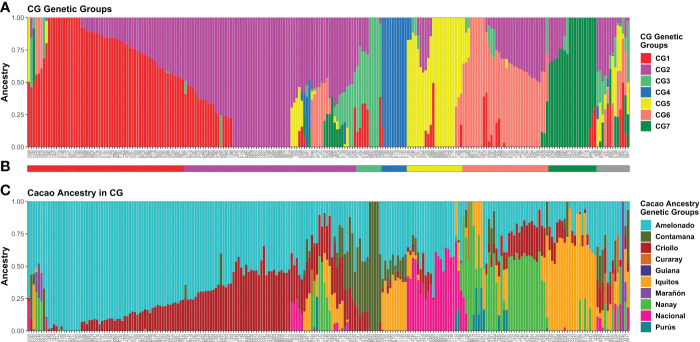
Memberships of 238 CG samples according to the ADMIXTURE program. Sample membership assuming *K* = 7 **(A)** as estimated by ADMIXTURE using cross-validation. Each column represents an individual. **(B)** Group assignment based on *K* = 7; Admixed plants (“Adm” group) in grey. **(C)** Membership to cacao ancestry genetic groups identified by Motamayor et al. (2008) using ADMIXTURE under supervised mode. Ancestry plot combining CG plants and cacao reference plants is shown in **Supplementary Figure S10**. Plots were generated using ggplot2 and ggpubr packages from the R program.

*Q*-matrices fulfilling the premise from *K* = 2 to *K* = 7 were analyzed with CLUMPAK (Kopelman et al., 2015). Among the *Q*-matrices included in the major clusters identified per *K* value, the ones belonging to the ADMIXTURE run showing the best combination of low CV error and low number of iterations to convergence for each *K* were selected for plotting purposes along with the membership matrix to the cacao ancestry genetic groups of Motamayor et al. (2008) (**Supplementary Figure S11**). Since a higher congruence was detected between *K* = 7 and the membership to cacao ancestry genetic groups, seven genetic groups (CG1–CG7) were assumed for CG plants (**Figure 3A**).

For plant assignment to the seven CG genetic groups (**Figure 3B**), all 212 individuals carrying *Q* > 0.5 to any given group were assigned accordingly. Another 13 plants with maximum *Q* < 0.5 and membership split into three groups were also allocated to the group with the highest *Q*. Finally, 13 samples were difficult to assign because they were highly mixed (membership to four or more groups, none with *Q* > 0.5) or presented a unique membership pattern. Thus, these plants were classified as Admixed (“Adm” group). Pure and mixed plants were detected in most groups after the assignment, except for CG4 (**Figure 3**).

**Figure 4:**
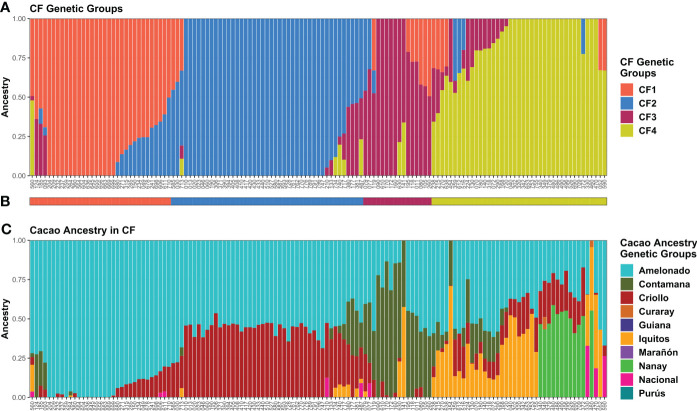
Memberships of CF samples according to the ADMIXTURE program. Sample membership assuming *K* = 4 **(A)** according to ADMIXTURE using cross-validation. Each column represents an individual. **(B)** Group assignment based on *K* = 4. **(C)** Membership to cacao ancestry genetic groups identified by Motamayor et al. (2008) using ADMIXTURE under supervised mode. Ancestry plot combining CF plants and cacao reference plants is shown in **Supplementary Figure S12**. Plots were generated using ggplot2 and ggpubr packages from the R program.

In cacao farm population structure analysis, the minimum at *K* = 4 in the CV error vs. *K* plot built from ADMIXTURE runs with the 135 CF samples strongly supported the presence of four groups (**Supplementary Figure S9B**), and all the groups had plants with high kinship (*Q* > 0.90). Thus, *K* = 4 was assumed as the most probable number of genetic groups (CF1–CF4) (**Figure 4A**). Plants carrying each possible combination of groups were detected in addition to the pure ones. CF plants were assigned to the group with the highest membership (**Figure 4B**). CF2 (45) was the largest group, and CF3 (16) was the smallest one.

Cacao genetic ancestries among CG and CF plants, according to Motamayor et al. (2008), shared some similarities. In both cases, Amelonado was the most abundant, with 49.22% in CG and 57.73% in CF of the total ancestry. Other common ancestries detected were Criollo (CG = 16.82% and CF = 19.13%), Iquitos (10.9% and 7.87%), Nanay (8.91% and 4.46%), and Contamana (6.51% and 9.83%). It excelled in the low representation of Nacional ancestry among CF samples in contrast to CG where it accounted for 5.36% of the total (**Figures 3C**, **4C**). Criollo and Nacional ancestries were only found in hybrid plants with Amelonado, and for the other common ancestries, both pure plants and hybrids were detected. Plants with high membership (*Q* > 0.9) to the Amelonado genetic group were identified in CG (30) and CF (21) while pure plants to Contamana (four), Iquitos (two), Nanay (one), and Marañon (one) were found in CG. Remarkably, this last one (C042) was located within the Admixed group, presumably because it was the unique individual showing a high *Q* to Marañon (**Figure 3**). Curaray, Purús, and Guiana ancestries were very low represented or absent among all studied plants. Each genetic group showed a distinctive cacao ancestry composition, and, in some cases, CG and CF groups were alike based on their cacao ancestries (**Figures 3**, **4**). In this sense, CG1 and CF1 were basically conformed by plants with high membership to Amelonado (CG1 average *Q* = 0.86, CF1 average *Q* = 0.91), slightly combined with other ancestries; CG2 and CF2 mostly contained hybrids of Amelonado and Criollo ancestries, though CG2 also had contributions from other ancestries; and finally, CG3 and CF3 showed almost pure Contamana individuals as well as combinations of Contamana mainly with Amelonado and Criollo.

Further similarities based on cacao ancestries between the remaining CG and CF genetic groups were difficult to establish because of their particular ancestry combination or proportion they carried. On one side, CG4 individuals were a complex mix of Amelonado, Iquitos, Contamana, and Criollo ancestries; CG5 group mainly contained hybrids of Amelonado and Nacional; CG6 had Nanay as distinctive ancestry alone or in combination with Amelonado, Criollo, and others; and CG7 had Iquitos individuals and their hybrids with Amelonado plus a portion of Criollo ancestry. On the other side, CF4 had the highest mixture of cacao ancestries among CF genetic groups, with plants carrying the combinations Amelonado–Iquitos–Contamana–Criollo, Amelonado–Nanay–Criollo, Amelonado–Iquitos–Criollo, and other minor combinations. Apparently, CF4 contained some of the ancestry combinations from CG4, CG6, and CG7, but the proportions were different; e.g., CG6 and CG7 plants had higher membership coefficients to Nanay and Iquitos, respectively, than CF4 plants carrying these ancestries (**Figures 3C**, **4C**). It was noteworthy that for CG and CF plants, the conformation of the genetic groups, as K increased from K=2 to the most probable K value (seven for CG and four for CF), was highly related to the putative origin of the plants based on their cacao ancestries (**Supplementary Figure S11** and **S13**).

Thus, using the model-based approach implemented in ADMIXTURE, seven and four genetic groups were detected among CG and CF samples, respectively. Cacao ancestry analysis revealed that some CG and CF groups had similar cacao ancestry compositions. Amelonado was the predominant ancestry in CG and CF plants; other commonly detected ancestries were Criollo, Contamana, Iquitos, and Nanay. Nacional ancestry was practically lacking in CF, while several CG plants had this ancestry as a hybrid with Amelonado. Marañon, Curaray, Purús, and Guiana ancestries were underrepresented or absent.

### Clustering and PCA of the Cuban cacao gene bank and cacao farms

**Figure 5:**
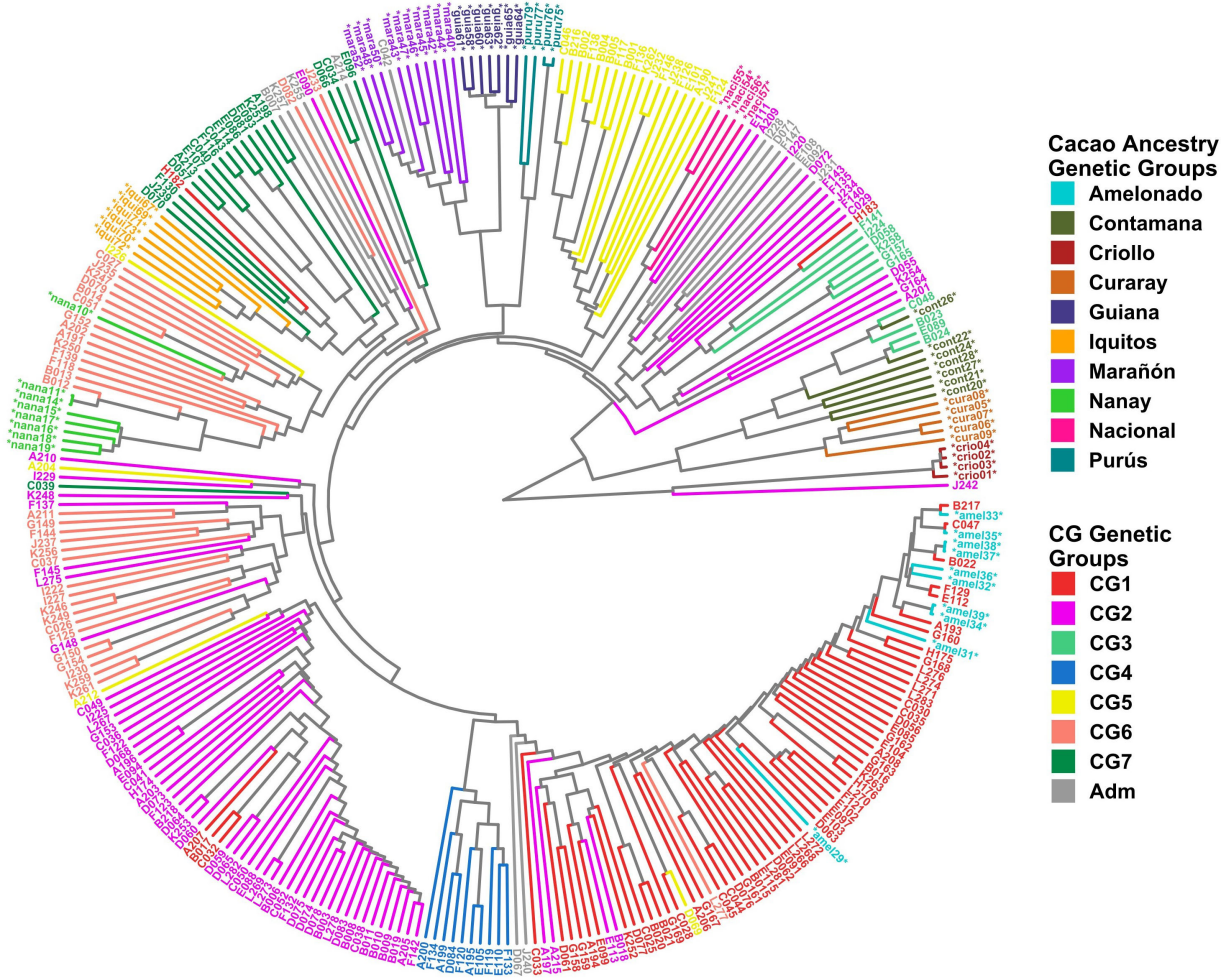
Dendrogram with 238 CG samples and 65 reference plants of cacao genetic groups. Clustering by UPGMA was based on a Hamming distance matrix calculated from a merge vcf file containing both sets of individuals with the 11,425 SNPs from the CG SNP dataset. Coloring is based on group membership from the ADMIXTURE program (*K* = 7) for the 238 CG samples (CG Genetic Groups) and cacao ancestry genetic groups according to Motamayor et al. (2008) for the 65 references (^*^). The plot was generated using ggtree and treeio packages from the R program.

**Figure 6:**
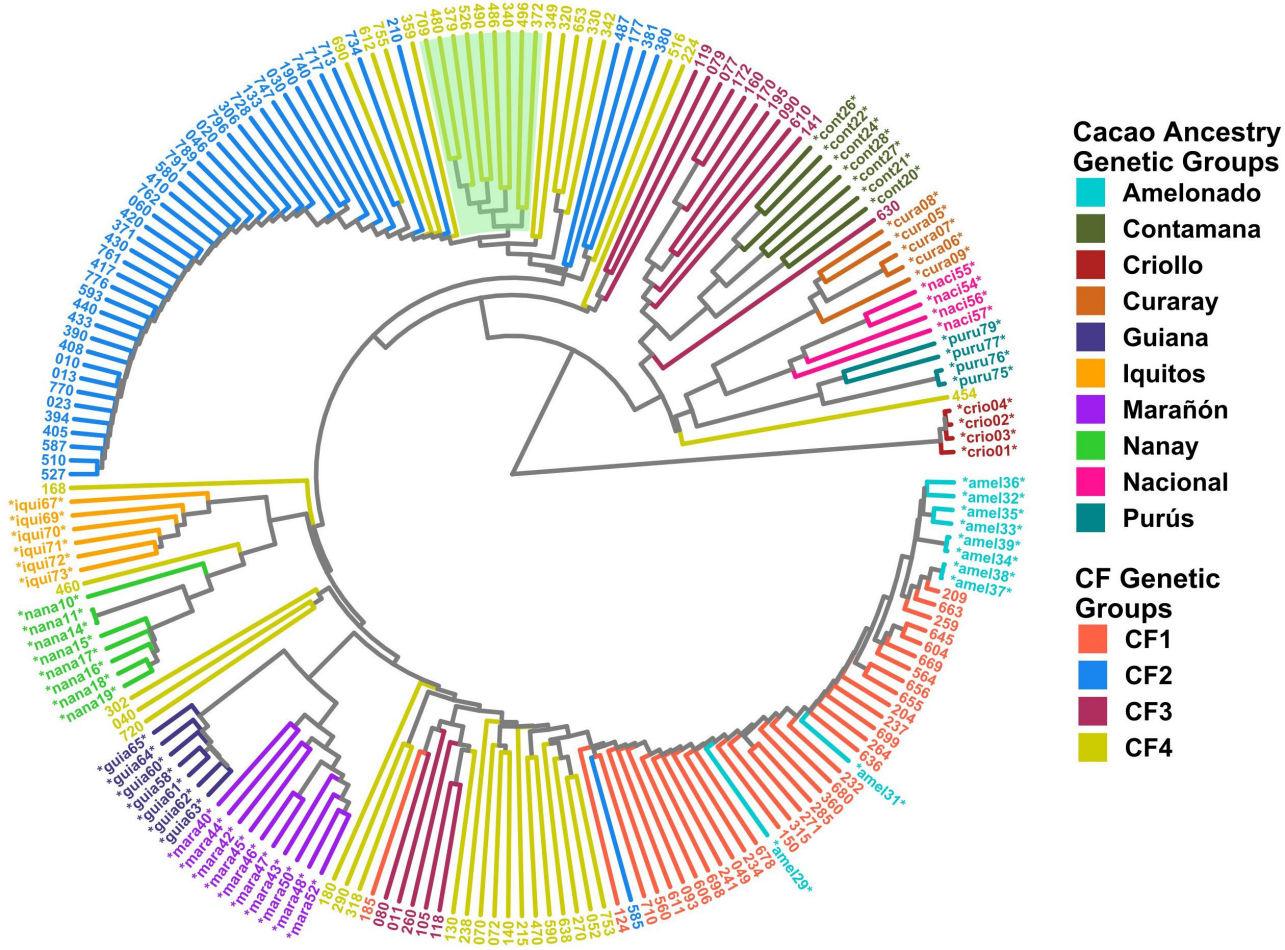
Dendrogram with 135 CF samples and 65 cacao genetic group controls. Clustering by the UPGMA method was based on a Hamming distance matrix calculated from a merge vcf file containing both sets of individuals with the 6,481 SNPs from the CF SNP dataset. Coloring is based on group membership from the ADMIXTURE program (*K* = 4) for the 135 CF samples (CF Genetic Groups) and cacao ancestry genetic groups according to Motamayor et al. (2008) for the 65 references (^*^). Light green background highlights hybrid individuals carrying Nanay ancestry. The plot was generated using ggtree and treeio packages from the R program.

Clustering analysis by UPGMA and PCA was used to confirm the genetic groups identified in CG and CF. Each analysis type was conducted with and without the 65 cacao plants used as a reference for the ancestry genetic groups. The dendrogram of either the 238 CG samples combined with the 65 references (**Figure 5**) or the 135 CF plants and the references (**Figure 6**) showed mostly congruent results with their respective population structure results. CG1, CG2, and CG4 plants, as well as CF1 and CF2, which all shared an important ancestry from Amelonado, basically formed independent clusters, which were allocated in the same branches as the reference plants of Amelonado in both dendrograms, though some plants from CG2 were located differently. Similarly, CG3 and CF3 samples carrying a high proportion of Contanama ancestry mainly clustered together in their respective dendrograms and closed to—or mixed with—Contamana reference plants (**Figures 5**, **6**).

A high congruence was also seen in CG5 (Nacional Ancestry) and CG7 (Iquitos) clustering, which mostly formed independent clades together with their respective cacao references (**Figure 5**). While CG6 (Nanay) were divided in two sub-groups, one of them laid next to the expected Nanay reference plants (**Figure 5**). CF4 individuals were also split in two major clusters (**Figure 6**), possibly as a result of their relatively high mix of cacao ancestry genetic groups revealed by ADMIXTURE (**Figure 4C**). Surprisingly, samples with Nanay ancestry within CF4 grouped together in the dendrogram (highlighted in green color, **Figure 6**), but apart from Nanay controls, which might be related to the lower Nanay ancestry of these individuals in comparison with CG6 plants. Interestingly, clustering analysis without cacao references improved the grouping of CG3 and CG6 individuals while having no effect on CF sample clustering (**Supplementary Figures S14, S15**). Finally, the dendrogram of CG samples revealed the proximity of C042 to the Marañon cacao reference plants (**Figure 5**), as opposite to the population structure analysis revealed by ADMIXTURE (**Figure 3**), which put C042 apart, although presenting 99% of ancestry to Marañon. This apparent discrepancy was due to the unique profile of C042 and the very low occurrence of Marañon ancestry in CG samples.

**Figure 7:**
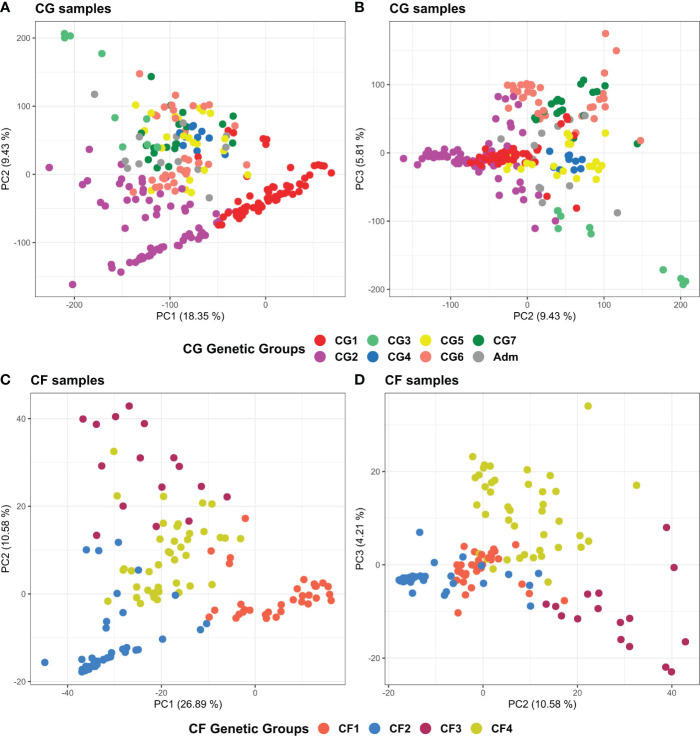
Principal component analysis plots of the 238 CG samples **(A, B)** and of the 135 CF samples **(C, D)** using CG and CF SNP datasets, respectively. **(A)** PC1 and PC2 of CG samples, **(B)** PC2 and PC3 of CG samples, **(C)** PC1 and PC2 of CF samples, and **(D)** PC2 and PC3 of CF samples. Coloring is based on group membership from the ADMIXTURE program, assuming *K* = 7 and *K* = 4 for CG and CF samples, respectively. Plots were generated using ggplot2 and ggpubr packages from the R program.

PCA results of the 238 CG and 135 CF plants mostly agreed with ADMIXTURE and clustering findings (**Figure 7**). The first three principal components explained 33.6% and 39.7% of the total variance of CG and CF SNP datasets, respectively. In both cases, PC1 and PC2 (**Figures 7A, C**) mostly separated genetic groups, carrying Amelonado (CG1, CF1) and Amelonado–Criollo hybrids (CG2, CF2) from each other and from the rest of the groups. However, several CG2 plants intruded into other groups, similar to the clustering results of CG2 (**Figure 5**), which would be associated with the presence of other ancestries apart from Amelonado and Criollo in these plants (**Figure 3**). PC2 and PC3 plots (**Figures 7B, D**) achieved the full separation of CG3 as well as CF3 and CF4. The other CG genetic groups were difficult to analyze because of the high mixed pattern observed, though CG6 and CG7 were mostly put apart from the rest of the samples without a clear separation between them. Nanay (found in CG6) and Iquitos (CG7) ancestries have proved to be difficult to separate from each other (Motamayor et al., 2008; Osorio-Guarin et al., 2017). PCA, combining either CG or CF samples with cacao reference plants (**Supplementary Figure S16**), put together CG1 and CF1 with Amelonado references as expected. Furthermore, most CG2 and CF2 plants, formed by Amelonado and Criollo hybrids, were mainly spread between Amelonado and Criollo reference plants, except for the CG2 plants carrying additional cacao ancestries. CG3 and CF3 plants were closed to Contamana references—or in between Amelonado and Contamana—and those plants with the highest membership coefficient to Contamana ancestry were the closet ones to these ancestry references. In the case of CF4, samples were related to different cacao ancestry genetic groups, as expected.

In general, clustering by UPGMA and PCA results were consistent with each other, and with population structure results supporting the presence of seven and four genetic groups among CG and CF plants, as well as the cacao ancestries identified in each genetic group. UPGMA performed better for CG samples than for CF, while PCA provided better support for CF genetic groups than for CG ones.

### Differentiation between groups and genetic diversity of the Cuban cacao gene bank and cacao farms

AMOVA and *F*_ST_ pairwise comparisons were independently performed on CG and CF plants to assess the genetic variability and differentiation among the identified groups. Similar contributions to variability were detected between CG (29.51%) and CF (30.15%) genetic groups, but the major contribution to the variability came from within groups with 70.49% (CG) and 69.85% (CF) of the total variance (**Tables 2**, **3**). For CF samples, farms and productive poles were also evaluated as putative sources of variation, but even less contribution to variability was detected using these levels (**Table 3**).

**Table 2:** AMOVA results from CG plants assuming seven genetic groups.

Source of variation	*Df*	SS	MS	Sigma	Variance (%)
Between groups	6	103,965.08	17,327.51	540.74	29.51
Within groups	218	281,633.74	1,291.90	1,291.90	70.49
Total	224	385,598.82	1,721.42	1,832.64	100.00

Df, degree of freedom; SS, square sum; MS, mean square. The Adm group was excluded from the analysis, p < 0.001.

**Table 3:** AMOVA results from CF samples assuming different organizing levels.

Source of variation	Identified CF groups			Cacao farms			Productive poles		
	*Df*	Sigma	Var (%)	*Df*	Sigma	Var (%)	*Df*	Sigma	Var (%)
Between organizing levels	3	293.83	30.15	6	60.15	6.65	2	46.92	5.14
Within organizing levels	131	680.68	69.85	128	844.88	93.35	132	865.58	94.86
Total	134	974.51	100.00	134	905.03	100.00	134	912.50	100.00

Organizing levels: identified groups with AMIXTURE software, cacao farms, and productive poles. Df, degree of freedom; Var, variance. Levels refer to the different hierarchy evaluated: identified groups (defined by ADMIXTURE), cacao farms (**Supplementary Table S1**), and productive poles (farm location: Jamal, San Luis and Paso de Cuba/Sabanilla), p < 0.001.

*F*_ST_ pairwise comparisons among either CG or CF genetic groups revealed all groups were significantly different (*p* = 0) from each other and supported a moderate to very large genetic differentiation (**Tables 4**, **5**). *F*_ST_ values for CG genetic groups (from 0.071 to 0.407) had a broader variation range than for CF groups (from 0.093 to 0.282). The highest *F*_ST_ values were obtained for the pairs CG1–CG3 (0.407) in CG and CF1–CF3 (0.282) in CF genetic groups. The cacao ancestries of the groups with the highest differentiation were alike, as CG1 and CF1 were mostly Amelonado plants, and CG3 and CF3’s distinctive ancestry was Contamana. On the other hand, the lowest *F*_ST_ values were estimated for the pairs CG6–CG7 (0.071) and CF2–CF4 (0.093) from CG and CF, respectively. Taking the groups individually, CG3 (*F*_ST_ ranging from 0.225 to 0.407) and CF1 (*F*_ST_ from 0.124 to 0.282) had the largest differentiation from the rest of the CG and CF genetic groups, respectively.

**Table 4:** *F*_ST_ pairwise comparison among the seven genetic groups identified in CG plants.

	CG1	CG2	CG3	CG4	CG5	CG6
CG2	0.144					
CG3	0.407	0.225				
CG4	0.243	0.174	0.279			
CG5	0.175	0.125	0.23	0.171		
CG6	0.213	0.142	0.252	0.162	0.129	
CG7	0.176	0.11	0.238	0.172	0.113	0.071

Adm samples were excluded from the analysis. All F_ST_ values were significant (p = 0).

**Table 5:** *F*_ST_ pairwise pairwise comparison among the four groups identified in CF plants.

	CF1	CF2	CF3
**CF2**	0.211		
**CF3**	0.282	0.192	
**CF4**	0.124	0.093	0.105

All F_ST_ values were significant (p = 0).

Genetic diversity parameters for all CG individuals (*H*_obs_ = 0.264, *H*_exp_ = 0.283, PIC = 0.235) were similar to the ones from CF plants (0.296, 0.286, 2.36) though *H*_obs_ was slightly higher for CF samples (**Table 6**). These parameters exhibited variability among both the CG and CF genetic groups. Genetic groups carrying the highest proportion of Amelonado ancestry (CG1 and CF1) had the lowest genetic diversity values. The highest *H*_obs_ were obtained in groups mostly conformed by Amelonado/Criollo hybrids (CG2 and CF2). CG6 and CG7 were very alike in terms of genetic diversity; these two groups had already shown the lowest *F*_ST_ values in group differentiation analysis. The CG4 group had a unique behavior since its *H*_exp_ (0.215) and PIC (0.162) values were remarkably lower than the estimated for CG samples, while *H*_obs_ (0.285) was slightly higher.

**Table 6:** Genetic diversity in CG and CF plants (total) and among CG and CF genetic groups.

	Group	*N*	*H*_obs_	*H*_exp_	PIC
**CG samples**	CG1	62	0.143	0.157	0.135
	CG2	68	0.346	0.299	0.234
	CG3	10	0.225	0.251	0.191
	CG4	10	0.285	0.215	0.162
	CG5	22	0.284	0.266	0.209
	CG6	34	0.295	0.276	0.224
	CG7	19	0.295	0.271	0.216
	**Total**	**225**	**0.264**	**0.283**	**0.235**
**CF samples**	CF1	33	0.114	0.129	0.115
	CF2	45	0.427	0.292	0.223
	CF3	16	0.296	0.305	0.236
	CF4	41	0.303	0.296	0.241
	**Total**	**135**	**0.296**	**0.286**	**0.236**

N, number of individuals; H_obs_, observed heterozygosity; H_exp_, expected heterozygosity; PIC, Polymorphic Information Content. Adm group was excluded from the analysis. The bold values represent the Total value of the parameters for CG and CF samples.

Summarizing, genetic groups from either CG or CF plants were significantly different from each other. Although genetic differentiation was higher among CG groups than among CF groups. The highest *F*_ST_ values came from pairwise comparison of groups carrying mostly Amelonado ancestry and those with Contamana background. Similar genetic diversity parameters were obtained in CG and CF samples. Genetic groups with the highest Amelonado ancestry proportion had the lowest genetic diversity parameters.

## Discussion

### SNP calling and SNP dataset characterization

Few protocols based on the double-digest approach of GBS technologies have been described for genetic studies in *Theobroma cacao*. Here, we used a ddRADseq approach (Peterson et al., 2012) with the enzymes *Eco*RI and *Nla*III to build three DNA sequencing libraries containing 406 samples from the Cuban cacao gene bank (CG) and cacao farms (CF). After data processing, the final number of SNPs identified in the CG (11,425 in 238 plants) and CF (6,481 in 135 plants) were higher than in other double-digestion-based GBS protocols used in cacao. Lachenaud et al. (2018) studied the population structure of 181 cacao clones from CIRAD’s Paracou-Combi station, French Guiana, with 3,409 SNPs derived from DArTseq technology based on a PstI/MseI genome digestion. Osorio-Guarin et al. (2020) used the enzyme combination BsaXI/CspCI to conduct the sequencing of 229 cacao accessions of Colombian germplasm collection and identified 8,131 or 9,003 SNPs depending on the reference genome used (Matina 1–6 or Criollo B97-61/B2, respectively). Adenet et al. (2020) assessed the cacao genetic diversity of 147 plants in Martinique and identified 4,113 SNP markers using GBS libraries built from double digestion with the same enzymes as Lachenaud et al. (2018).

The higher number of SNPs detected in our case is probably related to the technology and GBS protocol used. First, the combination of the enzymes NlaIII (four-cutter) and EcoRI (six-cutter) theoretically generates 81,158 DNA fragments between 300 bp and 500 bp from the Matina genome estimated by RADinitio software (Rivera-Colon et al., 2021). This number is higher than the 42,849 fragments predicted by the *in silico* Matina genome digestion with the enzyme combination described by Osorio-Guarin et al. (2020), even though they employed a broader fragment size selection (200–700). Second, the amount of DNA used (1 µg) in library preparation was larger than in other protocols (Adenet et al. (2020)—200 ng—and a greater amount of starting DNA helps in preventing biases during the PCR enrichment step, which could lead to genotyping errors during the SNP calling process (Andrews et al., 2016; Rivera-Colon et al., 2021). Last but not the least, the sequencing strategy we followed (150 bp, pair-end) should contribute to identifying more SNPs than the Osorio-Guarin et al. (2020) or Adenet et al. (2020) approaches, which used a 100-pb/pair-end and a 150-bp/single-end sequencing configuration, respectively. Interestingly, the number of filtered SNPs could be raised to 28,151 (CG) and 13,791 (CF) if the condition of SNP minimal separation of 1,000 base pairs during the VCFtools filtering step is removed.

A major goal of cacao genetic studies is to determine the presence of cacao genetic group ancestries in the plants under study, as described by Motamayor et al. (2008), which forces the use of cacao reference clones of those genetic groups. Two approaches have been reported to get the genotypes of the controls in GBS-based studies: 1° to process reference plants in the same way as the samples under study (Lachenaud et al., 2018) and 2° to exploit published data to get genotypes and hence the SNPs of a group of reference clones (Osorio-Guarin et al., 2020). We opted for the last choice and built a SNP dataset with 65 cacao reference plants, according to Cornejo et al. (2018) (**Supplementary Table S2**).

After intersecting the VCFtools-filtered SNPs with the cacao reference SNP dataset, 85.15% (11,425) of CG and 84.66% (6,481) of CF-filtered SNPs were retained. These percentages are higher than the ones reported by Osorio-Guarin et al. (2020), who obtained 3,712 SNPs out of 9,003 (45.65%) after intersecting their dataset with a SNP dataset built from raw sequence data of 69 cacao reference plants using Criollo cacao as reference genome. The capacity of the SNPs contained in both CG and CF SNP datasets to properly differentiate cacao genetic reference plants into 10 genetic groups was successfully confirmed (**Supplementary Tables S2–S6**; **Supplementary Figures S1–S6**) and validated the strategy followed.

The transitions/transversions ratio (Ti/Tv) estimated for the SNP datasets (1.647 for CG and 1.651 for CF) were similar to the 1.682 Ti/Tv value calculated for a cacao SNP dataset by Osorio-Guarin et al. (2018). This ratio has been used as a quality control parameter for checking the overall SNP quality during GBS experiments since SNP datasets of the same species should have similar values (Guo et al., 2014). The distribution of the per-SNP parameters used in GATK hard filtration showed the expected profile according to GATK hard filtering best practices (Caetano-Anolles, 2022). Put together, these results witness the overall good quality of the SNP datasets obtained for both sample groups.

The distribution of the SNPs along the cacao genome was different from that of Osorio-Guarin et al. (2020), who obtained the lowest and highest SNP density in chromosome 8 (25.13 per Mb) and chromosome 10 (32.15 per Mb), respectively. In our case, chromosome 1 had the highest average density, while chromosome 7 showed the lowest value for both CG and CF samples; the differences in the library preparation protocols (enzymes, fragment size) justify such behavior. We also detected an increase in SNP density as the locus moved from the chromosome center toward the telomeres. Such a pattern has already been described in GBS-based genetic studies of other crop species (Kumar et al., 2020; Yu et al., 2021). Centromeres and pericentromeric chromosome regions usually show a tendency of increased DNA methylation (Achrem et al., 2020); therefore, a lower amount of DNA fragments from this region should be expected when restriction enzymes sensitive to DNA methylation are used for genomic DNA digestion. EcoRI, one of the enzymes we used, is partially blocked by some combinations of overlapping in CpG-methylated DNA (NEB, New England Biolabs, 2022), which could support the lower number of SNPs detected toward the center of the chromosomes (**Figure 1**). On the other hand, many crop species such as barley, wheat, maize, tomato, and cotton showed high recombination rates in distal regions of the chromosome (Lloyd, 2022), which could favor the occurrence of genetic variation, including SNPs, in these parts of the genome.

The annotation of SNPs from both datasets located more than 83% of the variants in noncoding regions of the genome. Cornejo et al. (2018) also reported a large majority of the identified variants in noncoding regions using whole genome sequencing. However, the percentage of missense variants (average 9.4% for both datasets) and synonymous variants (~ 6.75%) that we obtained were higher than the 4.35% and 2.97% of missense and synonymous variants, respectively, obtained by these authors. This result supports a bias toward coding regions of the cacao genome with the ddRADseq sequencing protocol here described, likely associated with the enzyme combination used for genomic DNA digestion and the DNA fragment size selected.

Overrepresentation test results of genes comprising moderate and high-impact SNPs also revealed a bias since the gene molecular functions: protein kinase, ATP-related, and carbohydrate-binding activities were overrepresented while protein phosphorylation was the only biological process overrepresented. These aspects are common to many processes in plants, such as sensing, signaling, abiotic and biotic stress response, and growth, among others (Saijo and Loo, 2020; Chen et al., 2021). Therefore, these SNP datasets provided a suitable platform to deepen the genetic basis of physiological processes and agronomic indicators of cacao plants, in which the aforementioned functions and processes play a central role.

### Population analysis of the Cuban cacao gene bank and of cacao farms

The establishment of the Cuban cacao gene bank started more than 40 years ago and has been enriched throughout the years by solidary donations, the exchange of biological materials, and field expeditions. Presently, the collection hosts 282 cacao accessions, which constitute the genetic basis of the Cuban cacao improvement program and an important source of the genetic material used for cacao farming in the Baracoa region.

Using the model-based clustering of ADMIXTURE software, seven genetic groups were identified among the CG plants, while four were detected among cacao farm (CF) samples. The procedure we followed for best *K* value identification, including the premise that we set, helped in the proper detection of the genetic group number in CG as cross-validation (CV) error changes suggested no obvious *K* value. The success of CV error changes in best *K* identification depends in part on the degree of differentiation between the populations under study, as quantified by Wright’s fixation index, *F*_ST_ (Alexander and Lange, 2011). *F*_ST_ value for the CG6–CG7 pair was the lowest one (**Table 3**) among CG genetic groups, and since these groups were the last ones to be differentiated under *K* = 7 (**Supplementary Figure S11**), further group identification would be a difficult task. The number of genetic groups in cacao collections varies from one study to another: Boza et al. (2013) described nine lineages in a Dominican Republic collection revealed by 14 SSR markers; Osorio-Guarin et al. (2018) identified four groups within 565 clones in a CORPIOCA collection from Colombia using 96 SNPs; and three clusters were found in 133 Vietnamese cacao cultivars studied by a combination of SSR and SNP markers (Everaert et al., 2020).

Amelonado is the predominant ancestry among CG (49.22%) and CF (57.73%) plants, mostly as hybrids with the other cacao ancestries detected: Criollo, Contamana, Iquitos, Nacional, and Nanay. The prevalence of hybrids in cacao germplasm has been described in the collection from the Dominican Republic (Boza et al., 2013), Jamaica (Lindo et al., 2018), China (Wang et al., 2020), Vietnam (Everaert et al., 2020), Uganda (Gopaulchan et al., 2019), Nigeria (Olasupo et al., 2018), and others. However, groups contributing to hybrids are different; for instance, Amelonado contributed the most to collections in the Dominican Republic (51.7%) and China (59%), while Marañon is the more common ancestry in germplasm from Jamaica (29.9%) and Uganda (61.5% of the trees had ≥ 80% Marañon lineage). Amelonado/Criollo, Amelonado/Nacional, and Amelonado/Criollo/Nacional hybrids excelled among the identified lineages in CG because of their putative connection to the natural hybrids Trinitario and Refractario (Motilal, 2018). Trinitario clones are recognized for their high productivity and high cocoa quality, and Refractario constitutes a source of resistance to witches’ broom disease, a plague not reported in Cuba (Martínez de la Parte and Pérez Vicente, 2015) but detected in Caribbean islands close to Cuba (Evans, 2016; Ten Hoopen and Umaharan, 2017).

Cacao ancestry analysis in CF revealed Amelonado (CF1) and Amelonado/Criollo hybrid (CF2) plants as the most abundant (57.78%), which agrees with other in-farm cacao genetic diversity studies (Boza et al., 2013; Ji et al., 2013; Cosme et al., 2016; Adenet et al., 2020; Gopaulchan et al., 2020; Li et al., 2021) and the preference of farmers for planting grafted seedlings derived from putative Trinitario clones because of their better agronomic profile. Unfortunately, we did not detect pure Criollo plants in the CF plants as they were identified in farms from Honduras, Nicaragua, and Puerto Rico (Ji et al., 2013; Cosme et al., 2016). Another ancestry absent in Baracoa farm samples was Marañon, which has been identified in cacao fields in the Dominican Republic (Boza et al., 2013), Martinique (Adenet et al., 2020), Dominica (Gopaulchan et al., 2020), and Uganda (Gopaulchan et al., 2019).

Bidot Martínez et al. (2015) studied the population structure of anciently introduced cacao plants in Cuba, also known as traditional Cuban cacao. These are unique plants remaining within cacao farms from the central and eastern regions of the country. Some of the plants studied carried higher Criollo and Marañon proportions than those found in samples from cacao commercial farms in the Baracoa region (CF). Considering that only one plant from the CG had an important contribution from Marañon ancestry, these plants could represent an opportunity to increase the genetic resources available in the cacao gene bank for the strengthening of the Cuban cacao genetic improvement program.

Clustering analysis by UPGMA of CG and CF samples either alone (**Supplementary Figure S14, S15**) or combined with cacao reference plants (**Figures 5**, **6**) showed mostly congruent results with clustering by ADMIXTURE except for the CG2 and CF4 groups. Some CG2 individuals were located in different clades of the dendrogram apart from the main CG2 branch. Such behavior is probably a consequence of the relaxed rules followed for group assignment and the presence of several hybrid combinations within this group. The clustering of CF4 samples in a single branch with their expected cacao controls was difficult to achieve. The difficulties of clustering analysis to group Nanay and Iquitos hybrids from CF4 samples with their respective cacao ancestry genetic group references, as occurred with CG6 and CG7 samples (**Figure 5**), may be related to the lower membership coefficient of these CF individuals to Nanay (average *Q* = 0.4265) and Iquitos (0.4996), in comparison with the average memberships for Nanay (0.5767) and Iquitos (0.6665) of CG6 and CG67, respectively. The rest of the groups largely clustered together, with the expected cacao references confirming the results derived from ADMIXTURE software. On the other hand, PCA results of CG and CF plants showed adequate correspondence with ADMIXTURE and dendrogram results, though some CG genetic groups were not properly separated from each other in the PCA, but they were set apart from the rest of the groups.

### Differentiation between groups of the Cuban cacao gene bank and cacao farms

In spite of the small inconsistencies in the different approaches employed to assess population structure in CG and CF plants, the variability between the identified genetic groups revealed by AMOVA results was good enough to differentiate them, since all *F*_ST_ values from pairwise comparison concluded significant differences (*p* = 0). The low contribution to variability between levels when farms and productive poles were evaluated as sources of variation in AMOVA with CF samples suggests the same planting policies are followed in different regions in Baracoa, which in turn obey a national list of 15 cacao clones and eight related hybrids to be used in cacao farming (MINAGRI, 2019).

Group differentiation between CF genetic groups was less noticeable than CG genetic groups. The highest differences among CG and CF groups were detected between CG1–CG3 (0.407) and CF1–CF3 (0.282). CG1 and CF1 groups had Amelonado ancestry, while CG3 and CF3 contained Contamana backgrounds. Counting plants carrying a high membership coefficient (*Q* > 0.9) to any cacao ancestry revealed Amelonado (51) and Contamana (six, including two plants from CF3 with *Q* > 0.80) as the ancestries with the highest count of almost pure individuals among all CG and CF plants. Genetic groups containing plants with high membership coefficients to well-differentiated genetic backgrounds are expected to be the ones with the highest differentiation. The lowest differentiation in CG was achieved between CG6 and CG7 groups (0.071), which are mainly formed by hybrids of Nanay and Iquitos, respectively. Motamayor et al. (2008) have already recognized difficulties in the proper separation of Nanay and Iquitos groups in reduced diversity scenarios probably because individuals from these groups had been collected in a relatively small geographical area. Our results confirm those difficulties even though a large genetic differentiation (*F*_ST_ = 0.367) was detected between the reference plants of these cacao ancestry genetic groups using the same SNPs (**Supplementary Table S6**). The fact that we are working with hybrids instead of pure individuals would contribute to the low differentiation detected between the CG6 and CG7 samples. *F*_ST_ values for the rest of the pairwise comparisons varied from 0.105 to 0.407 and supported a moderate to very large differentiation between the groups (Frankham et al., 2002; Shu et al., 2021).

### Genetic diversity of the Cuban cacao gene bank and cacao farms

Genetic diversity statistics of CG plants showed moderate to low values of observed (*H*_obs_ = 0.264) and expected (*H*_exp_ = 0.283) heterozygosities and PIC (0.235), similar to the ones obtained for CF plants (0.296, 0.286, 0.236). Observed and expected heterozygosities from other cacao gene banks, estimated with SNP data, mostly had higher values. That is the case of Yunnan collection from China (*H*_obs_ = 0.361, *H*_exp_ = 0.306) (Wang et al. (2020), CORPIOCA collection in Colombia (0.353, 0.314) (Osorio-Guarin et al., 2017), germplasm bank of Tenguel-Guayas, Ecuador (0.479, 0.378) (Carranza et al., 2020), CRIG germplasm collection of Ghana (0.274, 0.343) (Takrama et al., 2014) and Uganda (0.304, 0.322) (Gopaulchan et al., 2019). Only the Jamaican collection had a lower *H*_exp_ (0.240), while *H*_obs_ (0.280) was again higher (Lindo et al., 2018). These cacao plants were genotyped with SNP markers selected for cacao classification. The selection was based on several SNP properties, such as level of polymorphism, distribution across the 10 cacao chromosomes, and SNP capacity to properly distinguish reference clones of cacao ancestry genetic groups (Ji et al., 2013; Fang et al., 2014; Motilal et al., 2017). Variant filtration based on polymorphic information was not applied to our SNP datasets. Therefore, lower genetic diversity statistics values should be expected on a per-locus basis, resulting from the random combination of low and high polymorphic SNP markers.

The within-group genetic diversity was also estimated among both CG and CF genetic groups. CG1 and CF1 had the lowest values for all genetic diversity parameters, while CG2 and CF2 showed the highest *H*_obs_ values. The lowest values of genetic diversity of CG1 and CF1 are consistent with the prevalence of Amelonado ancestry within this group. Amelonado and Criollo clones are recognized by their highly homozygous genomes and self-fertilization (Argout et al., 2011; Motamayor et al., 2013), justifying the low genetic diversity found in these groups. On the contrary, CG2 and CF2 excelled in the occurrence of Amelonado/Criollo hybrids and in the case of CG2 of other hybrid combinations (**Figures 3**, **4**). The crossing of highly homozygous Amelonado and Criollo plants should produce highly heterozygous Amelonado/Criollo hybrids, which, combined with the presence of other hybrids, could lead to the high values of CG2 and CF2 genetic diversity parameters, especially *H*_obs_. A high similarity was observed in CG6 and CG7 genetic diversity parameters. These results are consistent with low differentiation detected between these groups, as already discussed.

The genetic diversity of CG4 revealed some distinctions among the groups. This is a reduced, quite homogenous group of cacao plants with a unique mixture of cacao ancestries. Nine of its 10 individuals had a 0.9999 membership to this group (the last one was 0.8432), and cacao genetic group ancestries are split into Amelonado (0.4643), Iquitos (0.3689), Contamana (0.1355), and Criollo (0.0302). Genetic parameters of CG4 confirmed the observed singularities since the low *H*_exp_ and PIC obtained agree with the homogeneity of the individuals, and the cacao ancestries mix detected supports the excess of heterozygosity identified (*H*_obs_ > *H*_exp_).

### Final considerations

These results constitute the first attempt to use SNP markers in the assessment of the genetic diversity of cacao resources in the Baracoa region, which is responsible for most of the cacao production in Cuba. The cacao ancestry genetic group distributions among the CG and CF plants confirmed the poor utilization of diverse genetic groups in Cuban cacao farming, as also described in cacao agronomical practices worldwide (Zhang and Motilal, 2016; Cornejo et al., 2018). More cacao ancestries and ancestry combinations were detected among cacao gene bank accessions than in plants from commercial cacao farms of Baracoa. Therefore, there is a chance to increase the ancestries exploited in cacao production and, hence, the genetic diversity of the in-farm cacao resources using local cacao resources. To this end, cacao clones from the gene bank with ancestries different from those currently exploited in Baracoa cacao productive areas should be introduced into cacao farming practices. As an example, the results indicated a low representation of Nacional hybrids in cacao farms, but several clones of this type were detected in the germplasm collection. Refractario clones, which carry Nacional ancestry as hybrid clones, are known for their putative resistance to *Moniliophthora perniciosa* (witches’ broom disease), a disease absent in Cuba but present in Jamaica and the Dominican Republic. The introduction of cacao plants with a Nacional background in the productive areas of Baracoa may contribute to face future sanitary contingencies like the intrusion of witches’ broom disease from neighboring Caribbean Islands (Evans, 2016; Ten Hoopen and Umaharan, 2017).

Pure cacao plants belonging to the groups Amelonado, Contamana, Iquitos, Nanay, and Marañon were identified in the collection, but only one individual per group of the last two ancestries was found with *Q* > 0.9. Efforts should be made to increase the number of low-represented or absent ancestries such as Curaray, Guiana, and Purús in the Cuban national gene bank. Equally important would be the incorporation of pure individuals from the groups Criollo and Nacional, though several hybrids of these groups with Amelonado were identified. However, the population structure of the cacao collection accessions here described will contribute to the strengthening of the ongoing cacao improvement program by providing new approaches and revitalizing poorly exploited cacao clones present in the collection with attractive genetic backgrounds.

The high quality of the SNP datasets obtained, the genome widespread distributions of the variant sites, and the congruence among the results here presented to validate the use of the ddRADseq protocol described for genetic studies in *Theobroma cacao*. The exploitation of these datasets to conduct association studies could contribute to the identification of new genes and genome regions related to morpho-agronomic properties relevant to the cacao production. Particularly interesting in using RADseq in cacao is the potential identification of extrachromosomal SNPs (Laczkó et al., 2022). In our case, an average of 93% of the reads mapped to the 10 cacao chromosomes, leaving room for mitochondrial and plastid SNP identification. These aspects are still to be analyzed and could provide new ways for genetic studies in *Theobroma cacao*.

## Data availability statement

The datasets presented in this study can be found in online repositories. The names of the repository/repositories and accession number(s) can be found below: https://www.ebi.ac.uk/eva/, PRJEB71753.

## Author contributions

AR-R: Data curation, Formal analysis, Investigation, Methodology, Software, Validation, Visualization, Writing – original draft, Writing – review & editing. KM: Conceptualization, Investigation, Methodology, Writing – review & editing. MM-G: Investigation, Writing – review & editing. PC-: Investigation, Writing – review & editing. GE-L: Methodology, Writing – review & editing. IB-M: Conceptualization, Funding acquisition, Investigation, Project administration, Resources, Writing – review & editing. PB: Conceptualization, Funding acquisition, Investigation, Methodology, Project administration, Resources, Supervision, Writing – original draft, Writing – review & editing.
