# Supplementary material for: Using ddRADseq to assess the genetic diversity of in-farm and gene bank cacao resources in the Baracoa region, eastern Cuba, for use and conservation purposes
Source: Front Plant Sci. 2024 Mar 5;15:1367632. doi: 10.3389/fpls.2024.1367632 (PMC10948478; doi:10.3389/fpls.2024.1367632)
Supplement: Supplementary file 1 [file DataSheet_1.pdf]

Supplementary Material

1 Supplementary Data

2 Supplementary Figures and Tables

2.1 Supplementary Figures

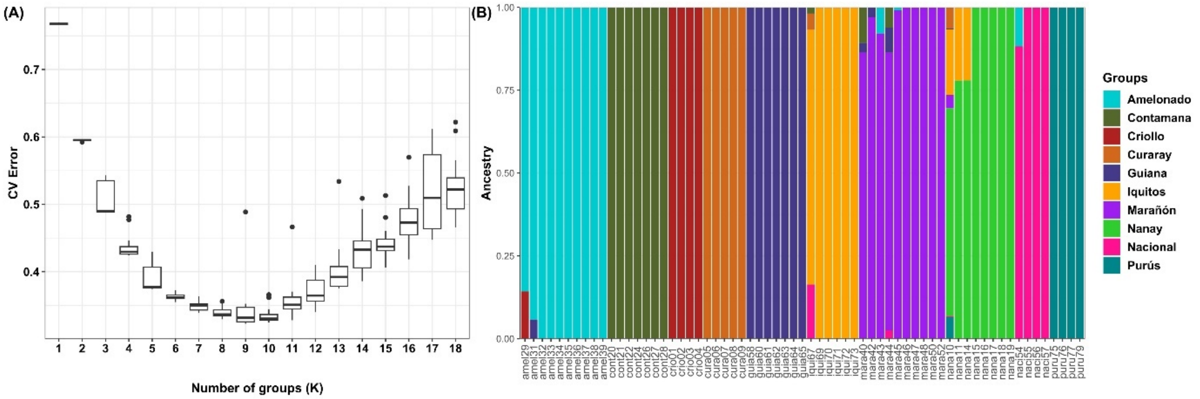

Supplementary Figure 1. Membership to cacao ancestry genetic groups of the 65 reference plants using the 11,425 SNPs from CG SNP dataset and ADMIXTUXE was run with the cross-validation procedure described in Material and Methods. (A) CV Error vs K plot for 20 independent ADMIXTURE runs per K; horizontal lines represent the median, boxes stand for the 25 and 75 % percentiles, vertical lines point to minimum and maximum values and the dots are “outlier” data. (B) Ancestry assuming K=10, the Q-matrix of ADMIXTURE run with the best combination of low CV error and low number of iterations to convergence was selected. Plot were generated using ggplot2 package from R program.

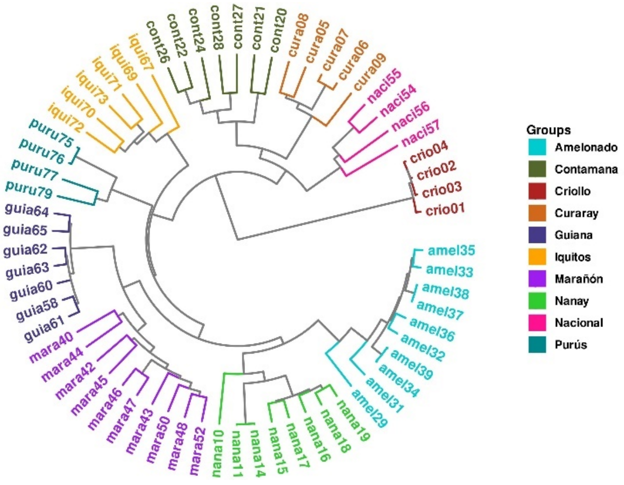

Supplementary Figure 2. Dendrograms of the 65 reference plants of cacao ancestry genetic groups using the 11,425 SNPs from CG SNP dataset. Clustering based on UPGMA from a Hamming distance matrix. Plot were generated using ggtree and treeio packages from R program.

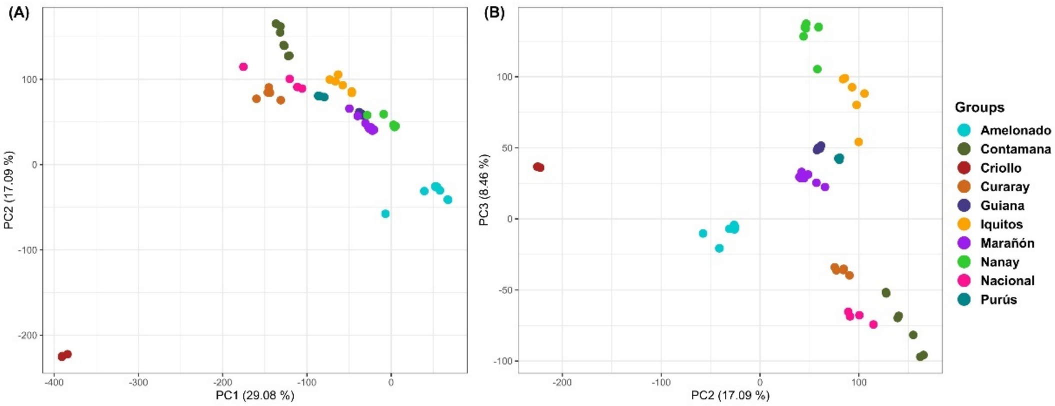

Supplementary Figure 3. Principal component analysis plots of the 65 reference plants of cacao ancestry genetic groups references using 11,425 SNPs from CG SNP dataset. (a) PC1 and PC3, (b) PC2 and PC3. Plots were generated using ggplot2 and ggpubr packages from R program.

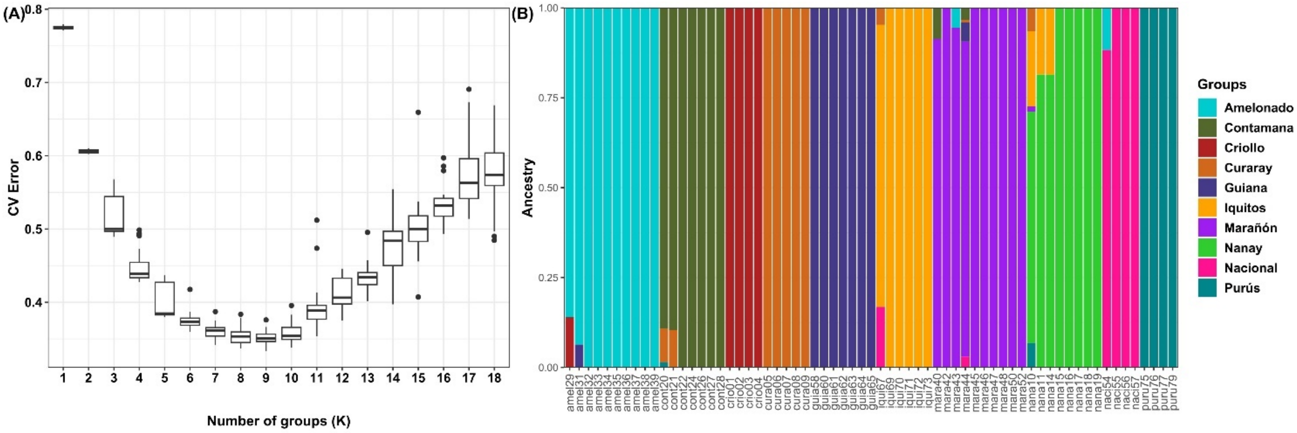

Supplementary Figure 4. Membership to cacao ancestry genetic groups of the 65 reference plants using the 6,481 SNPs from CF SNPs dataset. ADMIXTUXE was run with the cross-validation procedure described in Material and Methods. (A) CV Error vs K plot for 20 independent ADMIXTURE runs per K; horizontal lines represent the median, boxes stand for the 25 and 75 % percentiles, vertical lines point to minimum and maximum values and the dots are “outlier” data. (B) Ancestry assuming K=10, the Q-matrix of ADMIXTURE run with the best combination of low CV error and low number of iterations to convergence was selected. Plot were generated using ggplot2 package from R program.

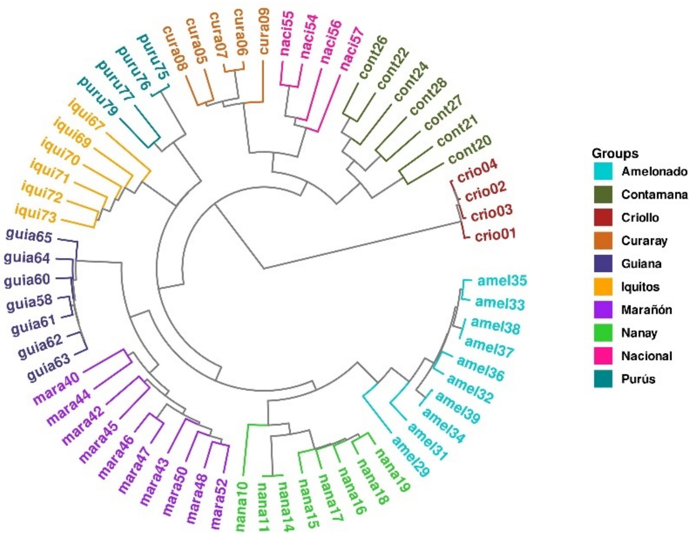

Supplementary Figure 5. Dendrograms of the 65 reference plants of cacao ancestry genetic groups using the 6,481 SNPs from CF SNPs dataset. Clustering based on UPGMA from a Hamming distance matrix. Plot were generated using ggtree and treeio packages from R program.

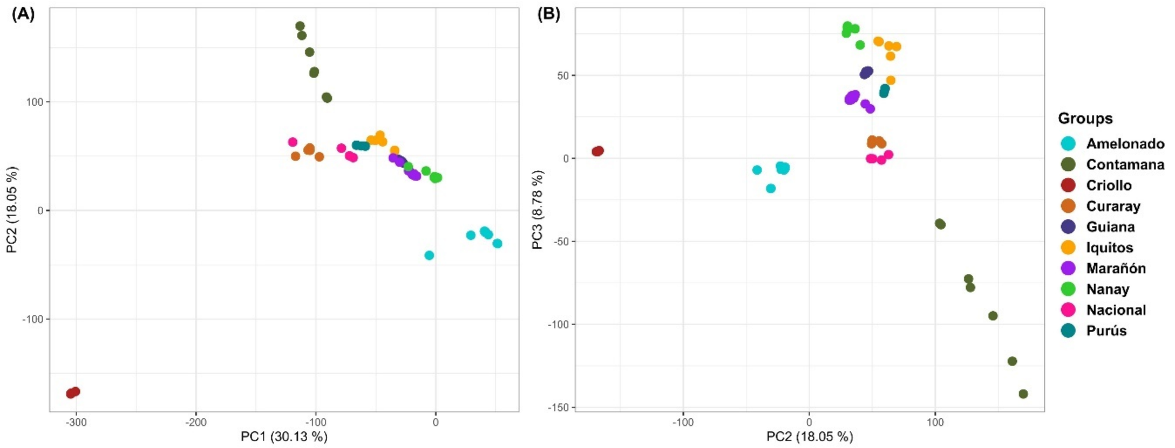

Supplementary Figure 6. Principal component analysis plots of the 65 reference plants of cacao ancestry genetic groups using the 6,481 SNPs from CF SNP dataset. (A) PC1 and PC3, (B) PC2 and PC3. Plots were generated using ggplot2 and ggpubr packages from R program

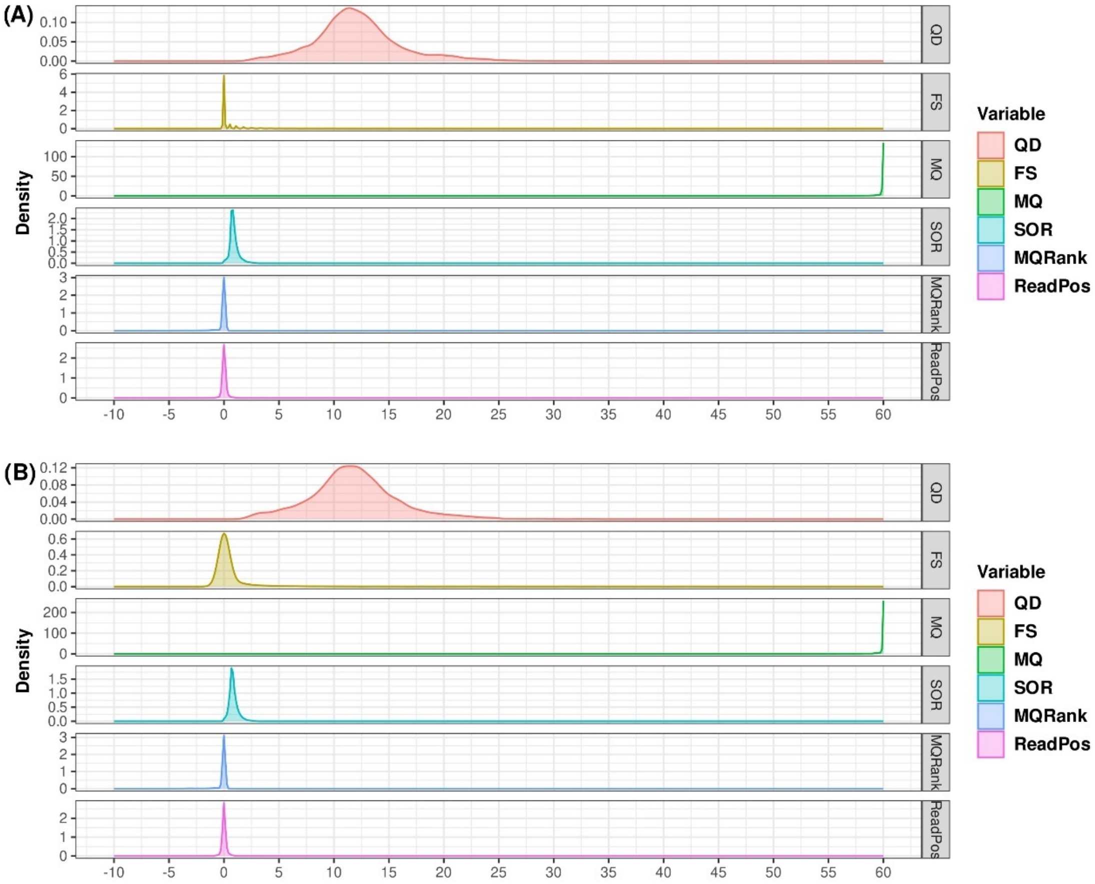

Supplementary Figure 7. SNPs quality check based on distribution of **QD** (*Quality by Depth*), **MQ** (*Mapping Quality*), **FS** (*Fisher Strand*), **SOR** (S*trand Odds Ratio*), **MQRank** (*Mapping Quality Rank Sum Test*) and **ReadPos** (*Read Position Rank Sum Test*) among the SNPs from CG (A) and CF (B) SNP datasets. Plots were built using ggplot and ggpurb R packages. **Note: QD** expected values are higher than 2 with peaks around 12 and 32. **FS**, **MQRank**, and **ReadPos** values closed to 0 suggest little or the absent of bias in the SNPs supporting data. **MQ** values around 60 (the maximum) are expected. **SOR** values should be between 0 and 3 (Caetano-Anolles, 2022).

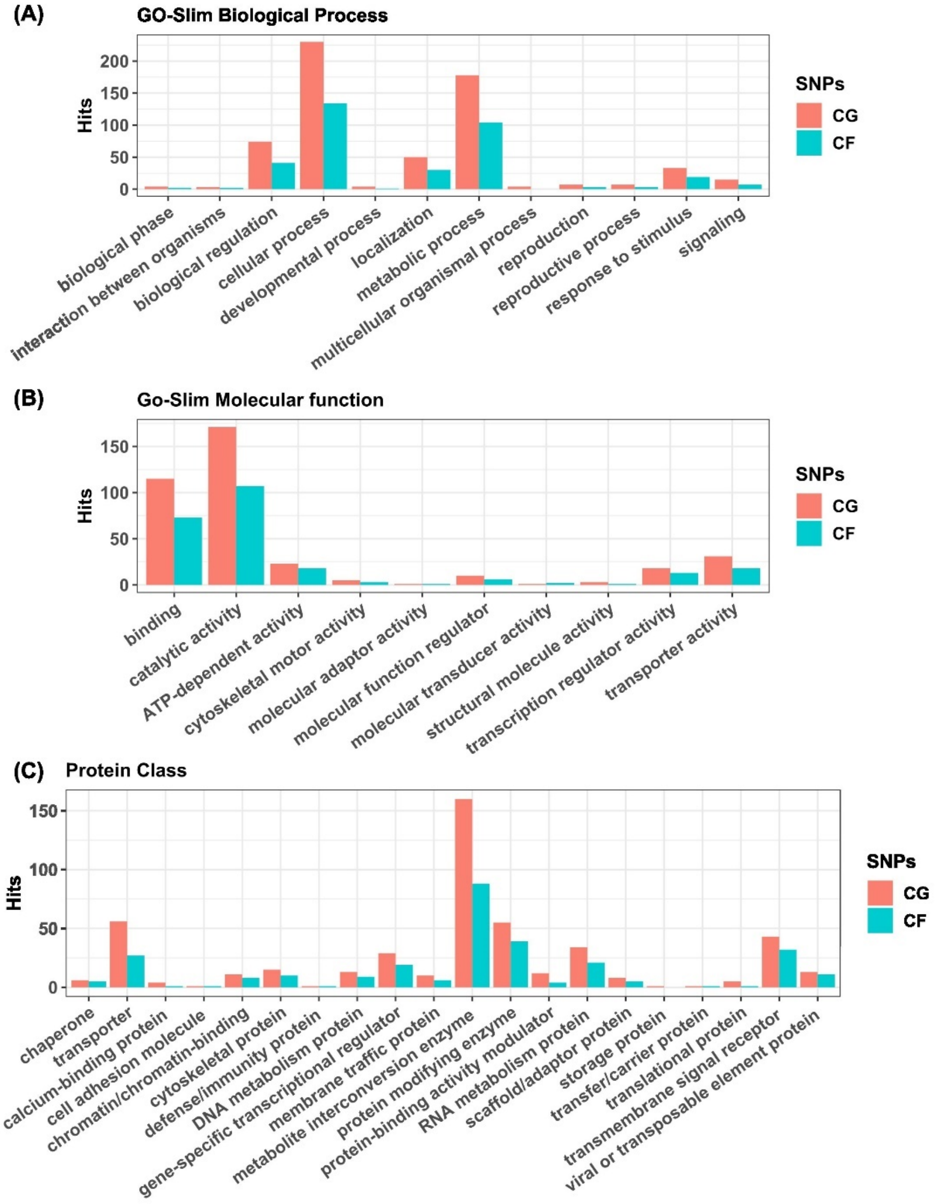

Supplementary Figure 8. Results of gene ontology analysis using PANTHER classification system based on gene list built from CG and CF SNP datasets annotation. Gene list contained genes carrying SNPs with moderate or high impact according to SnpEff. Hit counting with terms or categories from databases GO-Slim biological process (A), GO-Slim molecular function (B) and Protein class (C) are shown.

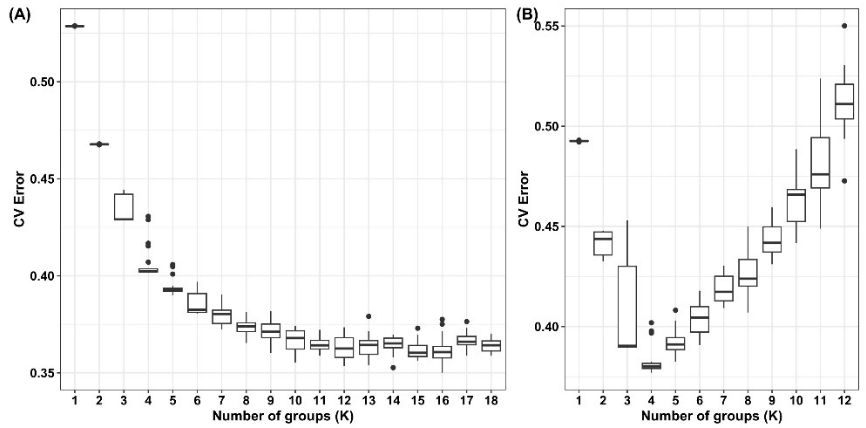

Supplementary Figure 9. Cross validation error (CV Error) vs number of groups (K) plots for 20 independent ADMIXTURE runs per K using the 11,425 and 6,481 SNPs from CG (A) and CF (B) samples, respectively. Horizontal lines represent the median, boxes stand for the 25 and 75 % percentiles, vertical lines point to minimum and maximum values and dots are "outlier" data.

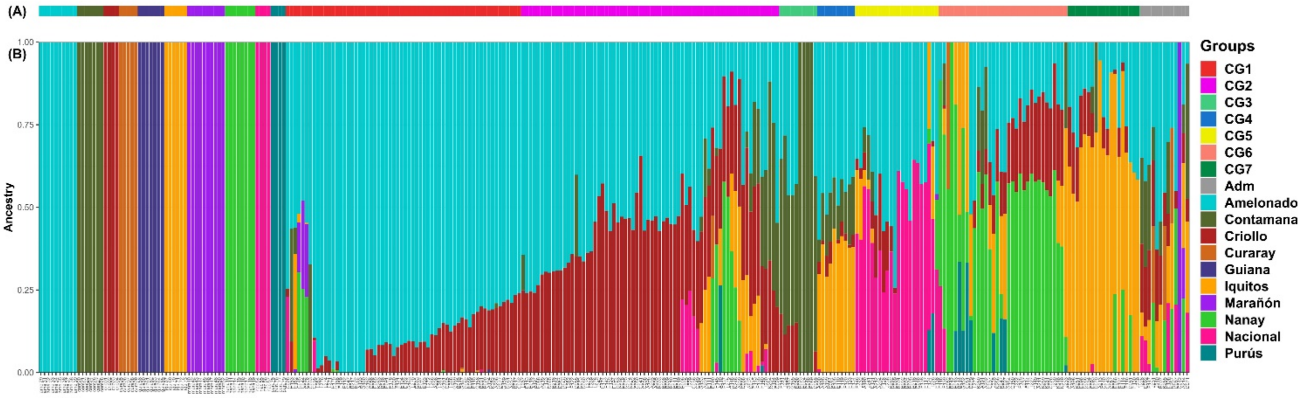

Supplementary Figure 10. Membership to cacao genetic groups of 238 CG plants and 65 reference plants of cacao ancestry genetic groups assuming K=10. ADMIXTURE was run under supervised mode using the 11,425 SNPs from CG SNP dataset. (A) Assignment to cacao ancestry genetic groups or CG genetic groups identified by ADMIXTURE (Figure 3, main text). (B) Membership to the cacao genetic groups identified by Motamayor *et al.* (2008). Plot was generated using ggplot2 and ggpubr packages from R program.

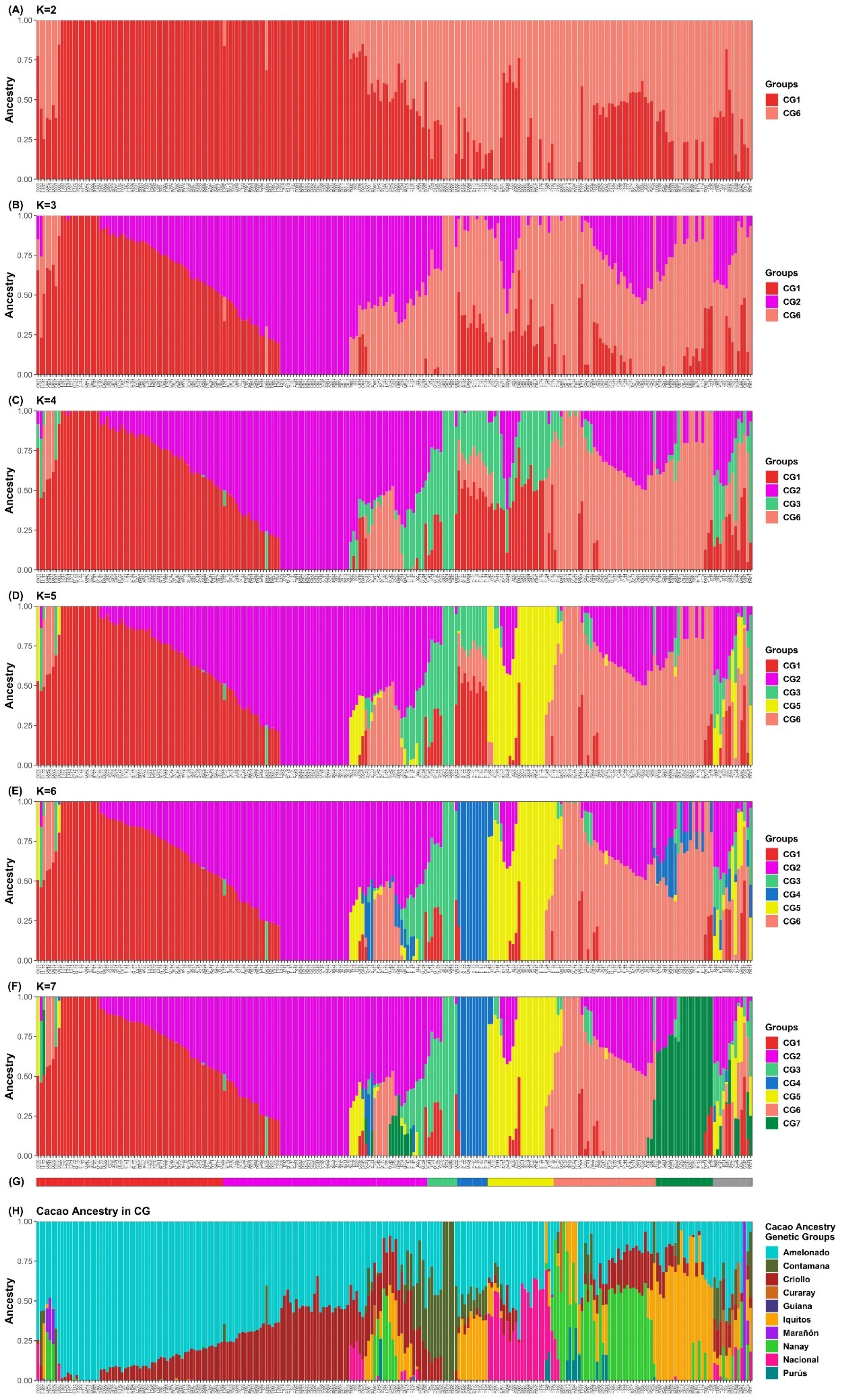
(A) K=2
(B) K=3
(C) K=4
(D) K=5
(E) K=6
(F) K=7
(G)
(H) Cacao Ancestry in CG
Ancestry
1.00
0.75
0.50
0.25
0.00
Groups
CG1
CG2
CG3
CG4
CG5
CG6
CG7
Cacao Ancestry Genetic Groups
Amelonado
Contamana
Criollo
Curaray
Guiana
Iquitos
Marañón
Nanay
Nacional
Purús

Supplementary Figure 11 (Previous page). Memberships of 238 CG samples according to ADMIXTURE program. Samples membership assuming K=2 (A), K=3 (B), K=4 (C), K=5 (D), K=6 (E) and K=7 (F) as estimated by ADMIXTURE using cross validation. Each column represents an individual. (G) Group assignment based on K=7, Admixed plants ("Adm" group) in grey. (H) Membership to cacao ancestry genetic groups identified by Motamayor *et al.* (2008) using ADMIXTURE under supervised mode. Ancestry plot combining CG plants and cacao reference plants is shown in Supplementary Figure 10. Plots were generated using ggplot2 and ggpubr packages from R program.

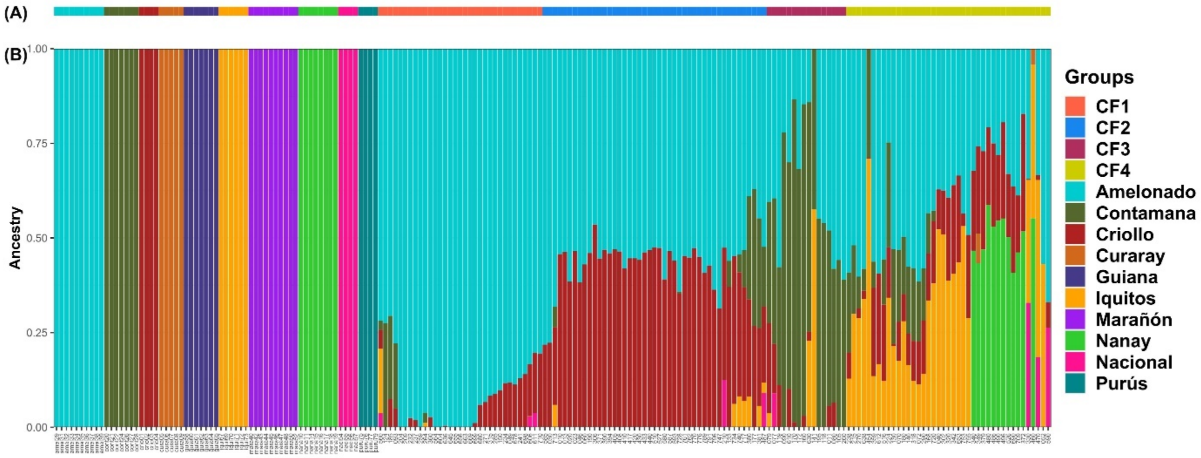

Supplementary Figure 12. Membership to cacao genetic groups of 135 CF plants and 65 reference plants of cacao ancestry genetic groups assuming K=10. ADMIXTURE was run under supervised mode using the 6,481 SNPs from CF SNP dataset. (A) Assignation to cacao genetic groups or CF groups identified by ADMIXTURE (Figure 6, main text). (B) Membership to the cacao genetic groups identified by Motamayor *et al.* (2008). Plot was generated using ggplot2 and ggpubr packages from R program.

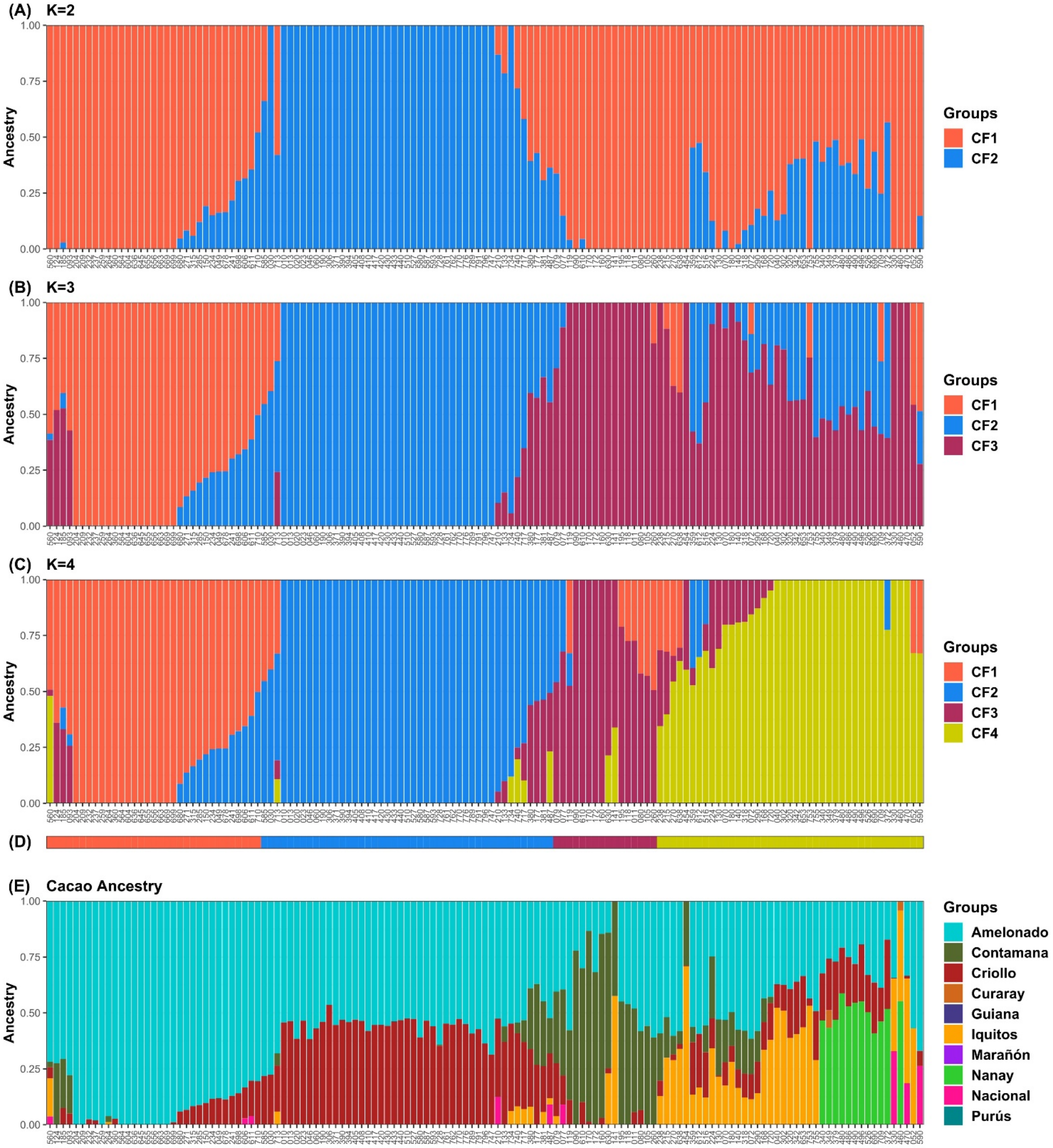

Supplementary Figure 13. Memberships of CF samples according to ADMIXTURE program. Samples membership assuming K=2 (A), K=3 (B) and K=4 (C) according to ADMIXTURE using cross validation. Each column represents an individual. (D) Group assignment based on K=4. (E) Membership to cacao ancestry genetic groups identified by Motamayor *et al.* (2008) using ADMIXTURE under supervised mode. Ancestry plot combining CG plants and cacao reference plants is shown in Supplementary Figure 13. Plots were generated using ggplot2 and ggpubr packages from R program.

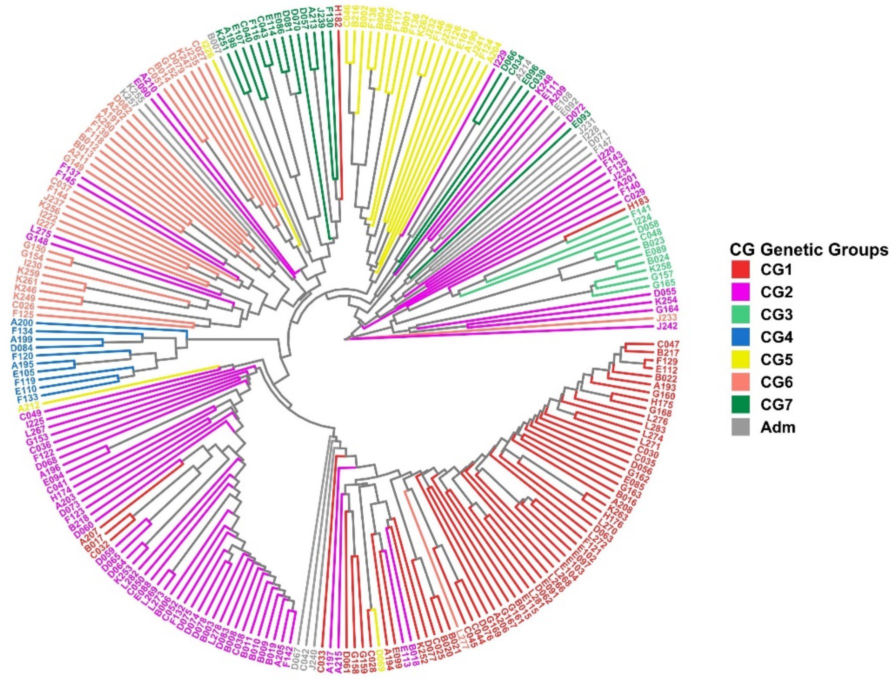

Supplementary Figure 14. Dendrogram with 238 CG samples. Clustering by UPGMA from a Hamming distance matrix based on the 11,425 SNPs from CG SNP dataset. CG plants coloring is based on the membership assuming K=7 from ADMIXTURE program. Plot was generated using ggtree and treeio packages from R program.

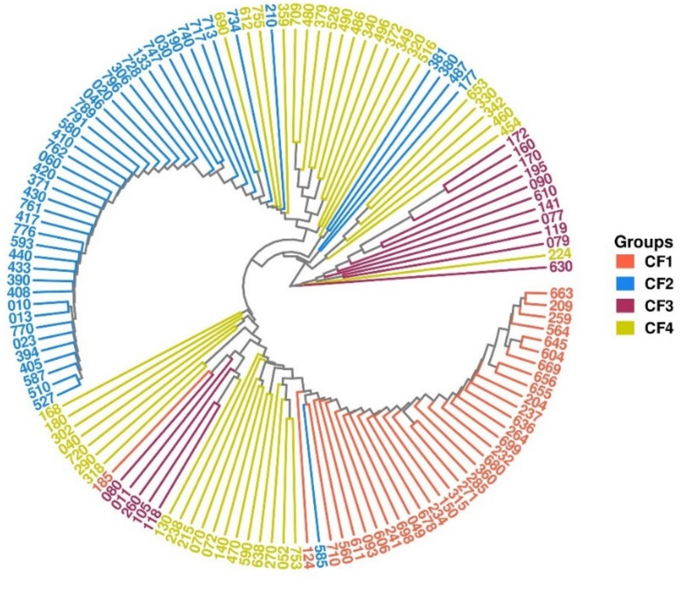

Supplementary Figure 15. Dendrogram with 135 cacao farms samples. Clustering by UPGMA from a Hamming distance based on the 6,481 SNPs from CF SNP dataset. CF individual coloring is based on the membership assuming K=4 from ADMIXTURE program. Plot was generated using ggtree and treeio packages from R program.

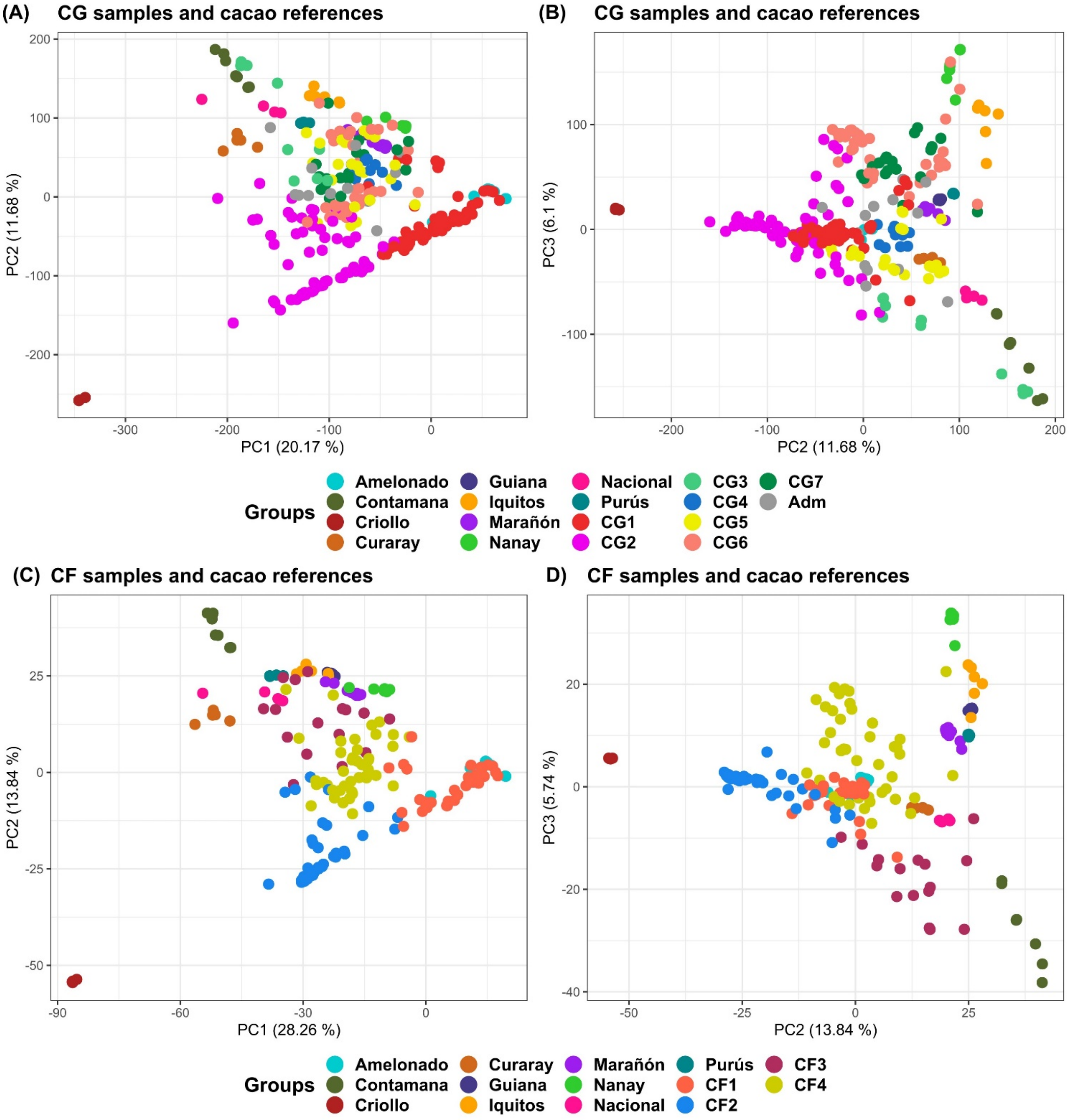

Supplementary Figure 16. Principal component analysis plots of the 238 CG samples and 65 cacao genetic groups references using the 11,481 SNPs from CC SNPs dataset (A and B) and the 135 CF samples and 65 cacao genetic groups references using the 6,481 SNPs from CF SNPs dataset (C and D). (A) PC1 and PC2 of CG and reference plants, (B) PC2 and PC3 PC2 of CG and reference plants. (A) PC1 and PC of CF and reference plants, (B) PC2 and PC3 of CF and reference plants. Samples coloring is based on membership assuming K=7 for CG samples, membership assuming K=4 for CF samples and cacao ancestry genetic groups for reference plants. Plots were generated using ggplot2 and ggpubr packages from R program.

2.2 Supplementary Tables

Supplementary Table 1. Details of the cacao farms used for sampling purpose.

Farm ID	Productive Pole	Farm Name	Farm Type	Cacao plants origin	North Coordinate	West Coordinate	Farm Size (ha)	Plots (Unit)	Sampled Plant (Unit)
F08	Jamal	Finca Los Yaser	4	Traditional / Hybrid / Grafted	20°16,413' N	74°25,644' W	16	8	40
F15	Jamal	Santa Rita	4	Traditional / Hybrid / Grafted	20°16,638' N	74°25,521' W	9,45	5	25
F02	San Luis	Finca Santa María	3	Traditional / Hybrid	20°18,730' N	74°25,610' W	11,7	3	15
F19	San Luis	La Esperanza I	2	Grafted	20°17,698' N	74°26,779' W	3	3	15
F05	Paso de Cuba / Sabanilla	Finca Elcita	1	Hybrid / Grafted	20°17,101' N	74°27,907' W	5,33	3	15
F10	Paso de Cuba / Sabanilla	Finca San Miguel	1	Hybrid / Grafted	20°15,577' N	74°27,713' W	6,66	6	30
F11	Paso de Cuba / Sabanilla	Poca Pena	2	Grafted	20°15,192' N	74°27,625' W	8,5	4	20

Note: Farm type as described in Materials and Methods. **Farm size** refers to total amount of hectare of the farm not only to the cacao planted area. **Plot** is number of plots raised in the cacao plantation. **Sampled Plant** refers to the number of plants collected for analysis purposes in the farm**.**

Supplementary Table 2. List of clones used as references plants of cacao ancestry genetic groups according to Cornejo *et al.*, (2018).

ID	Plant Code	Genetic Group
crio01	Criollo	Criollo
crio02	Sp1	
crio03	Sp3	
crio04	Sp9	
cura05	Cur 3 G39 - A10	Curaray
cura06	Cur 3 G37 - A6	
cura07	Cur 3 G38 - A8	
cura08	LCTEEN - 141	
cura09	SIL -1-G56-A6	
nana10	SPEC - 194 75	Nanay
nana11	Pound 7	
nana14	Pound 7B	
nana15	Pound 10-B	
nana16	NA - 92	
nana17	NA - 331	
nana18	NA - 286	
nana19	NA - 702	
cont20	NH - 53	Contamana
cont21	NH - 40	
cont22	T 695 -SCA6 - A1	
cont24	SCA 24.2	
cont26	SCA -11	
cont27	PMF - 27	
cont28	PMF - 20	
amel29	TRD86	Amelonado
amel31	REDAMEL 1-31	
amel32	SIAL 84	
amel33	SIAL 70	
amel34	SIC 806	
amel35	mvP30	
amel36	SIAL 169	
amel37	Matina	
amel38	Matina Tica 2	
amel39	Catongo	

ID	Plant Code	Genetic Group
mara40	PA – 218	Marañón
mara42	PA – 150	
mara43	PA – 51	
mara44	PA – 107	
mara45	PA – 169	
mara46	MO – 4	
mara47	MO – 9	
mara48	PA 289	
mara50	PA -56	
mara52	PA -121	
naci54	UF 273 T1	Nacional
naci55	UF 273 T2	
naci56	AM -1 -54	
naci57	Brisas -1	
guia58	GU - 308A	Guiana
guia60	GU -300 P	
guia61	GU – 222	
guia62	GU - 291F	
guia63	GU - 175P	
guia64	GU - 114P	
guia65	GU - 255V	
iqui67	IMC – 51	Iquitos
iqui69	IMC – 12	
iqui70	IMC – 50	
iqui71	IMC – 20	
iqui72	IMC -67	
iqui73	IMC – 14	
puru75	CAB 77 - PL5	Purús
puru76	CAB 76 - PL3	
puru77	RB 47 - PL3	
puru79	RB 39 - PL1	

Legend: ID: Identifier used in this study, **Plant Code:** Code as Cornejo *et al.* (2018), **Genetic Group:** Plant memberships to cacao ancestry genetic groups defined by Motamayor *et al.* (2008) and assigned by Cornejo *et al.* (2018). **Note:** Sequence data were downloaded from NCBI (*BioProject* PRJNA486011) using SRA toolkit v2.11.0 (SRA Toolkit Development Team, 2022) and processed as described (Cornejo *et al.*, 2018). The identified SNPs were confirmed with the SNP list deposited in European Variation Archive (https://www.ebi.ac.uk/eva/, *Project accession code* PRJEB28591).

Supplementary Table 3. AMOVA results of reference plants of cacao ancestry genetic groups using the 11,425 SNP from of CG SNP dataset.

Source of Variation	Df	SS	MS	Sigma	Variance (%)
Between groups	9	131,092.75	14,565.86	2,167.56	76.76
Within groups	55	36,102.11	656.40	656.40	23.24
Total	64	167,194.86	2,612.42	2,823.97	100.00

Legend: Df: Degree of freedom, **SS:** Square Sum, **MS:** Mean Square. Highly significant values with $p < 0.001$.

Supplementary Table 4. Fst pairwise comparison among the reference plants of cacao ancestry genetic groups using the 11,425 SNPs from CG SNP dataset.

	Amel	Cont	Crio	Cura	Guia	Iqui	Mara	Nana	Naci
**Cont**	0.709								
**Crio**	0.923	0.773							
**Cura**	0.794	0.456	0.824						
**Guia**	0.749	0.630	0.926	0.750					
**Iqui**	0.558	0.433	0.762	0.501	0.550				
**Mara**	0.478	0.486	0.790	0.567	0.401	0.379			
**Nana**	0.608	0.625	0.872	0.705	0.676	0.367	0.474		
**Naci**	0.712	0.414	0.796	0.457	0.681	0.412	0.502	0.636	
**Puru**	0.718	0.447	0.846	0.568	0.659	0.381	0.429	0.579	0.504

Legend:
Amel: Amelonado
Cont: Contamana
Crio: Criollo
Cura: Curaray
Guia: Guiana
Iqui: Iquitos
Mara: Marañón
Nana: Nanay
Naci: Nacional
Puru: Purús

Note: All Fst values were significant (p=0).

Supplementary Table 5. AMOVA results of the reference plants of cacao ancestry genetic groups using the 6,481 SNPs of CF SNP datasets.

Source of Variation	Df	SS	MS	Sigma	Variance (%)
Between Groups	9	70,074.10	7,786.01	1,158.79	76.81
Within Groups	55	19,246.08	349.93	349.93	23.19
Total	64	89,320.17	1,395.63	1,508.72	100.00

Legend: Df: Degree of freedom, **SS:** Square Sum, **MS:** Mean Square. Highly significant values with $p < 0.001$.

Supplementary Table 6. Fst pairwise comparison among the 65 reference plants of cacao ancestry genetic groups reference plants using the 6,841 SNPs from CF SNP dataset.

	Amel	Cont	Crio	Cura	Guia	Iqui	Mara	Nana	Naci
**Cont**	0.711								
**Crio**	0.926	0.769							
**Cura**	0.800	0.465	0.834						
**Guia**	0.756	0.621	0.927	0.749					
**Iqui**	0.582	0.450	0.782	0.516	0.568				
**Mara**	0.487	0.495	0.797	0.568	0.393	0.387			
**Nana**	0.620	0.626	0.883	0.713	0.687	0.370	0.477		
**Naci**	0.698	0.394	0.801	0.451	0.658	0.396	0.463	0.617	
**Puru**	0.725	0.445	0.852	0.582	0.661	0.389	0.426	0.585	0.478

Legend:
Amel: Amelonado
Cont: Contamana
Crio: Criollo
Cura: Curaray
Guia: Guiana
Iqui: Iquitos
Mara: Marañón
Nana: Nanay
Naci: Nacional
Puru: Purús

Note: All Fst values were significant (p=0).

Supplementary Table 7. Transition and transversion statistics in CG and CF SNP datasets.

**Change (Type)**	**Cacao Gene Bank**		**Cacao Farms**	
	**Count**	**%**	**Count**	**%**
Total SNPs	11,425	100	6,481	100
C/T	3,608	31.58	1,995	30.78
A/G	3,501	30.64	2,041	31.49
**Ts**	**7,109**		**4,036**	
C/G	765	6.70	457	7.05
G/T	1,138	9.96	634	9.78
A/C	1,112	9.73	620	9.57
A/T	1,301	11.39	734	11.33
**Tv**	**4,316**		**2,445**	
**Ts/Tv**	**1.647**		**1.651**	

Legend: Change: Refers to the substitution type, **Count:** numbers of changes, **C:** Cytosine, **T:** Thymine, **A:** Adenine, **G:** Guanine, **Ts:** Transitions, **Tv:** Transversions, **Ts/Tv:** Transitions/Transversions ratio.

Supplementary Table 8. Q-matrices matching the high membership premise for each K value assessed with CG samples.

K	No of ADMIXTURE runs for each K value	Q-matrices matching the premise	Q-matrices no matching the premise
2	20	20	0
3	20	20	0
4	20	20	0
5	20	19	1
6	20	17	3
7	20	9	11
8	20	3	17
9	20	4	16
10	20	2	18
11	20	0	20
12	20	0	20
13	20	0	20
14	20	0	20
15	20	0	20
16	20	0	20
17	20	0	20
18	20	0	20
